# A Current Averaging Strategy for Maximizing Analyte and Minimizing Redox Interference Signals with Square Wave Voltammetry

**DOI:** 10.1021/acs.analchem.4c01053

**Published:** 2024-05-26

**Authors:** Katherine J. Levey, Julie V. Macpherson

**Affiliations:** Department of Chemistry, University of Warwick, Coventry CV4 7AL, U.K.

## Abstract

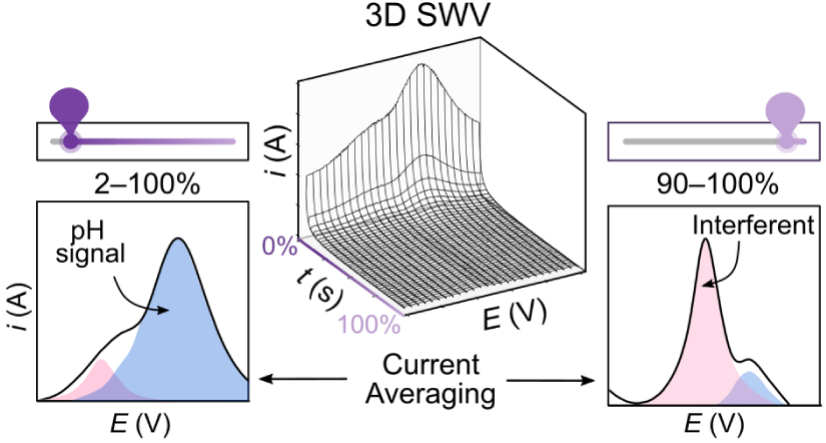

Square wave voltammetry (SWV) is commonly used in electroanalytical applications to enhance analyte faradaic signals and minimize nonfaradaic processes. However, little attention is given as to how best use SWV to minimize faradaic interference signals that arise from redox species present in solution that have redox potentials that convolute with that of the analyte. In conventional SWV, a series of current–time (*i*–*t*) transients are collected, and *i* is averaged over a specified window of each transient (potentiostat dependent). This average *i* is reported against the electrode potential, *E*. As the *i*–*t* response is governed by the type of electron transfer reaction under investigation, we show how by collecting all *i*–*t* data and through judicious choice of the current averaging window, it is possible to enhance the analyte response while at the same time reducing the interferent signal. We look at three different electron transfer reactions, fast electron transfer outer sphere, metal electrodeposition/stripping, and surface-confined proton-coupled electron transfer (PCET) and demonstrate different *i*–*t* behaviors in SWV, visually aided by the use of 3D *i*–*t*–*E* plots. In the case of PCET quinone-based voltammetric sensing of pH in the presence of a heavy metal (here Cu^2+^), we show that the use of a much earlier current averaging window (2–10% of the *i*–*t* response) results in the pH signal being clearly distinguished from that of the overlapping heavy metal.

## Introduction

For the detection of target analytes in electroanalytical applications such as biomolecule sensing^1−3^ and environmental monitoring,^4,5^ pulsed voltammetric techniques offer higher sensitivity and lower detection limits than cyclic voltammetry (CV).^6^ A variety of different waveforms can be used for the application of the potential pulses, leading to a range of techniques, such as normal pulsed voltammetry,^7^ differential pulsed voltammetry,^8^ and perhaps the most commonly employed, square wave voltammetry (SWV).^6,9^

**Figure 1:**
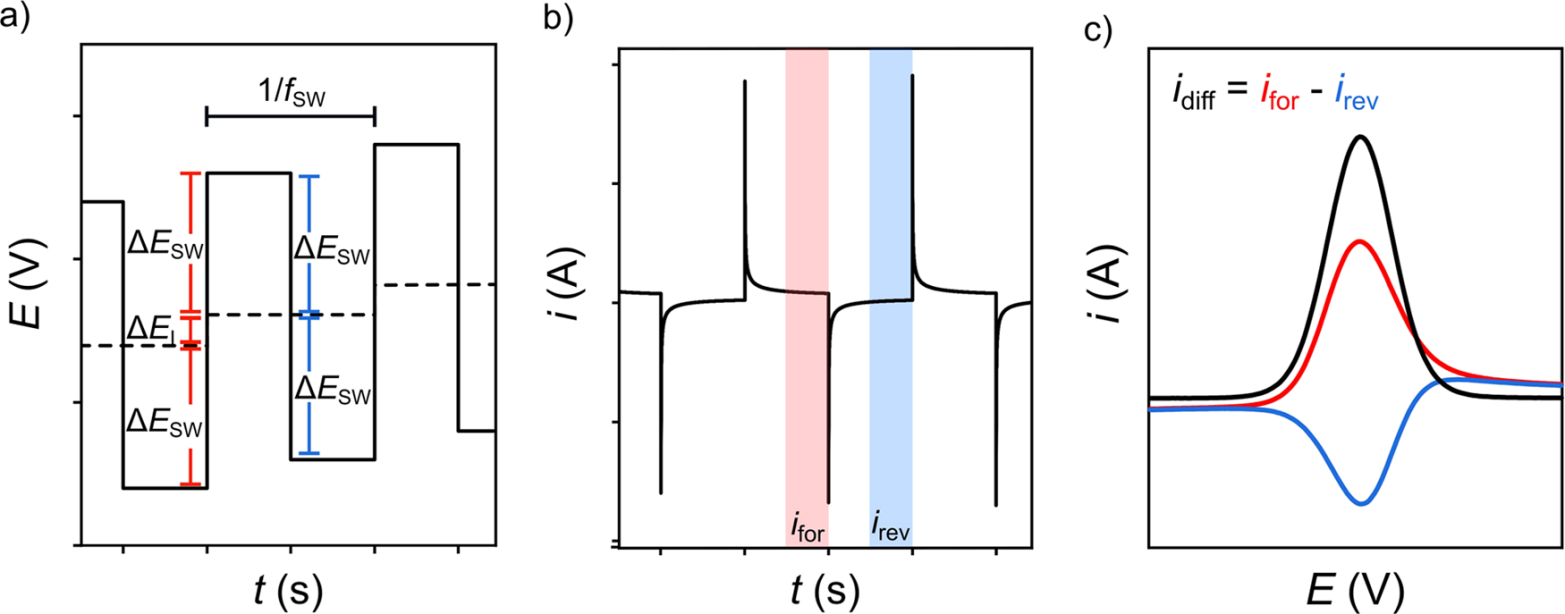
(a) Schematic of the potential waveform (solid) including the linear potential ramp (dashed), which is the *E* reported in SWV and Δ*E*_I_, Δ*E*_SW_, and *f*_SW_. (b) Schematic of the resulting *i*_for_ and *i*_rev_–*t* transients. The highlighted red and blue areas represent a current averaging window of 50–100%. (c) Plots of *i*_for_ (red), *i*_rev_ (blue), and *i*_diff_ (black) 2D-SWV curves.

The technique of SWV is built upon the early square wave polarography work by Barker and colleagues,^10,11^ but SWV as it is known today was first described by Ramaley and Krause,^9^ and then later developed by the Osteryoungs.^12,13^ In a conventional CV, the waveform is a linear staircase potential ramp with a defined potential step height, Δ*E*_I_ (dashed line in Figure 1a). In SWV, the linear ramp of a CV trace is overlaid with a square wave pulse of amplitude ± Δ*E*_SW_, as shown in Figure 1a (solid line). This SWV pulse sequence results in a series of current–time (*i*–*t*) curves (Figure 1b), with the forward current measured over the entire forward pulse potential (=2Δ*E*_SW_ + Δ*E*_I_, labeled in red in Figure 1a) and the reverse current measured over 2Δ*E*_SW_ (labeled in blue in Figure 1a). As such, the SWV waveform is defined by Δ*E*_I_, Δ*E*_SW_, and the frequency, *f*_SW_, which determines the time taken, τ, for each full cycle (τ = 1/*f*_SW_), Figure 1a.

The current reported for each forward and reverse potential pulse (Figure 1b) can be sampled by taking a single point from the end of each forward (*i*_for_) and reverse (*i*_rev_) *i*–*t* transient. The difference between these current values is reported as a difference current (*i*_diff_ = *i*_for_ – *i*_rev_). However, rather than using a single point at the end of the pulse, most commercial potentiostats are designed to average the current across a defined window of the pulse, which we refer to as the current averaging window. This does vary between manufacturers (see * additional note after Acknowledgements at the end of the paper) but is typically at least the last half ≥50–100% (Figure 1b) of the *i*–*t* pulse, where 100% is the end of the pulse. Most potentiostats simply output the sampled (and averaged) currents by reporting *i*_diff_ as a function of the staircase potential, *E*, *i.e., i*_diff_–*E* (black line in Figure 1c), which we herein refer to as a 2D SWV. Note, *i*_for_–*E* (red line) and *i*_rev_–*E* (blue line) are also usually accessible (Figure 1c). Provided that electrolyte solutions are conductive enough, and *f*_SW_ is not too high, it is possible to sample the current from regions of the *i*_diff_–*t* traces that are free from electrical double-layer charging.^6,9,12^ In this way, background charging currents can be eliminated enabling the faradaic current to be fully resolved.^9,12^

Within the literature, changing SWV parameters such as *f*_SW_, Δ*E*_SW_, and Δ*E*_I_ has provided a route to further optimize and obtain additional information on redox systems.^14,15^ For example, White and Plaxco demonstrated how *f*_SW_ could be tuned accordingly to enhance the SWV current associated with either the unbound or target-bound response of an electrochemical aptamer-based sensor.^2^ Further work by Plaxco and coworkers showed the advantages of making SWV measurements at a second *f*_SW_, where the current was constant irrespective of aptamer binding, in order to produce a calibration-free sensor.^16^ A significant body of work by Mirčeski and coworkers considered how by varying Δ*E*_SW_^17,18^ and Δ*E*_I_,^15^ it was possible to gain mechanistic insight into the electron transfer kinetics of a variety of different electron transfer reactions, including more complex electrocatalytic and anodic stripping reactions.^19,20^

While changing SWV operating parameters is one route for gaining additional insight and control of electrochemical processes, there is a wealth of data lost by only using 2D (*i*_diff_–*E*) SWV data. Cobb and Macpherson demonstrated how further analytical information, such as electrolyte conductivity could be obtained by analyzing each individual *i*_for_ and *i*_rev_ transient of the SWV waveform.^21^ Abeykoon and White later showed how it was possible to extract SWV data for a multitude of different frequencies in one SWV scan by undertaking analysis of the entire series of *i*_for_ and *i*_rev_ transients. This resulted in significant time advantages compared to changing *f*_SW_ systematically.^22^ Mirčeski and coworkers used repetitive chronoamperometric measurements, instead of collecting continuous *i*–*t* curves as in the work of Macpherson and Cobb^21^ and Abeykoon and White,^22^ to characterize electrode reaction mechanisms.^23^

To date, little work has been done which shows how by examining the *i*–*t* traces in full as a function of *E*, for different classes of electron transfer reaction, e.g., reversible, quasi-reversible, surface-bound redox, metal electrodeposition/stripping, it is possible to determine SWV parameters, which enhance or diminish current signals. This is particularly important when there are interfering redox signals present in a voltammetric potential window, which can often be the case when examining real-life water systems in electroanalytical applications. However, such an approach leads to the generation of significant data for analysis. It is thus informative to be able to visualize the data in such a way that can help inform which data sets are the most useful for analysis or predict the most useful SWV operating conditions. We propose the use of 3D SWV where the *i*_diff_*–t* data for each *E* value is represented in the form of a 3D plot. While 3D visualization of SWV data is relatively new, for CV analysis, 3D representation was first shown in the 1960s.^24^ Since then sampled current voltammetry has also been studied in 3D to investigate the electron transfer kinetics of inner sphere and surface-bound redox processes at different electrode materials.^25,26^

In this paper, we show how 3D SWV can be used as a guide to help understand and deconvolute the 2D SWV response of two redox species in solution, which have similar detection potentials; in particular, solution pH (the analyte) via proton-coupled electron transfer (PCET) of surface-bound quinone groups from that of the heavy metal Cu^2+^ (the interferent). From 3D SWV plots, the most appropriate current averaging window for the *i*_diff_–*t* versus *E* transients can be established, which enhances the analyte signal while at the same time suppressing the redox-active interferent signal.

## Experimental Section

### Materials and Solutions

Milli-Q water (Millipore Corp., resistivity 18.2 MΩ cm at 25 °C) was used to prepare all solutions. For unbuffered measurements, 0.1 M potassium nitrate (KNO_3_, ≥99.0%, Honeywell) was adjusted with 0.1 M potassium hydroxide (KOH, ≥85%, Sigma-Aldrich) and 0.1 M sulfuric acid (H_2_SO_4_, Fischer Scientific) to change the pH to the desired value. Solution pH and conductivity were measured using a glass pH probe (PHC301, Hach Company) that was calibrated using pH 4, 7, and 10, powder buffer pillows (Hach Company). Ferrocenylmethyltrimethylammonium hexafluorophosphate (FcTMAPF_6_, made in-house),^27^ and copper(II) sulfate pentahydrate (CuSO_4_(H_2_O)_5_, ≥98%, Fischer Scientific) were used in experiments.

### Electrode Fabrication

#### Boron-Doped Diamond (BDD)

BDD disks, 3 mm in diameter (*d*) and ∼600 μm thick, were cut from a freestanding BDD wafer (Electroanalytical grade,^28^ Element Six, Oxford) using a 355 nm Nd:YAG 34 ns laser micromachining system (E-355H-ATHI-O system, Oxford Lasers). The growth (electrochemical) face was polished to <10 nm root-mean-square (RMS) roughness and the nucleation face lapped to ∼1 μm RMS. All BDD disks were acid cleaned using concentrated H_2_SO_4_ (98%, Fischer Scientific) and KNO_3_ (0.75 g per ml),^5^ to remove any loose sp^2^ carbon generated during laser micromachining.

#### Quinone Functionalized BDD (BDD-Q)

The BDD growth face was laser micromachined with a series of concentric rings, 15.5 μm wide and spaced 50 μm apart, using five laser passes and a fluence of 429 J/cm^2^; this converts the BDD surface to a robust form of sp^2^ carbon.^29^ Optical images of the BDD-Q electrodes are shown in Supporting Information 1, Figure S1.

After acid cleaning, the backside of the BDD(-Q) electrodes were sputtered with a Ti/Au (10:400 nm) ohmic contact (Moorfield MiniLab 060) and then annealed with the growth face facing downward at 400 °C for 5 h. A 3 mm brass rod was contacted to each electrode with silver epoxy (Chemtronics, CircuitWorks), and the assembly heated in an oven for 1 h at 60 °C. The brass rods were pushed into a 3D printed electrode body (Form 3, 3D printer using UV cure resin, Tough 2000, FormLabs) that was prefilled with the same resin.^30^ The electrodes were partially UV cured for 10 min with a UV lamp then fully cured for 30 min at 60 °C using the Form Cure system (Formlabs). The electrodes were polished using silicon carbide paper (Buehler) and alumina paste (MicroPolish suspension, 0.05 μm, Buehler) on a sheet of alumina to expose the electrode surface, followed by rinsing with distilled water.

### Electrochemical Setup

The BDD, BDD-Q, or polycrystalline gold (Au) disk (*d* = 2 mm, CH Instruments) electrodes were used with a Ag/AgCl reference electrode (3 M KCl, Radiometer Analytical) and a Pt coil counter electrode. The AutoLab PGSTAT128N potentiostat (Metrohm) with the ADC10 M ultrafast sampling and SCAN250 modules was employed for all electrochemical measurements at *T* = 21 °C. SWV measurements utilized Δ*E*_SW_ = 50 mV, *f*_SW_ = 25 Hz, and Δ*E*_I_ = 4 mV and a quiet time of 2 s. Each step of the linear ramp across a 1 V vs Ag/AgCl potential window contained forward and reverse pulses, each 20 ms in length. Using an *f*_SW_ of 25 Hz enables the current to be sampled every 10 μs (10 kHz), while keeping the total number of data points within the ∼10^6^ maximum points of the onboard memory of the instrument. The bandwidth of the control amplifier loop was set to the maximum of 1 MHz to prevent the bandwidth from influencing electrochemical processes at early time points (∼10's μs). To record the full *i*–*t* trace generated during a SWV measurement, the “Chrono Methods” technique (AutoLab PGSTAT128N) was utilized. This allows for a series of potential steps to be input by the user and recorded in one continuous measurement. The data output is a continuous series of 502 *i*–*t* transients ∼10 s in total duration. Alternative methods for recording the raw SWV *i*–*t* data are possible, e.g., a LabView-based method has been employed with a CH Instrument potentiostat.^22^

### 3D SWV Representation

**Figure 2:**
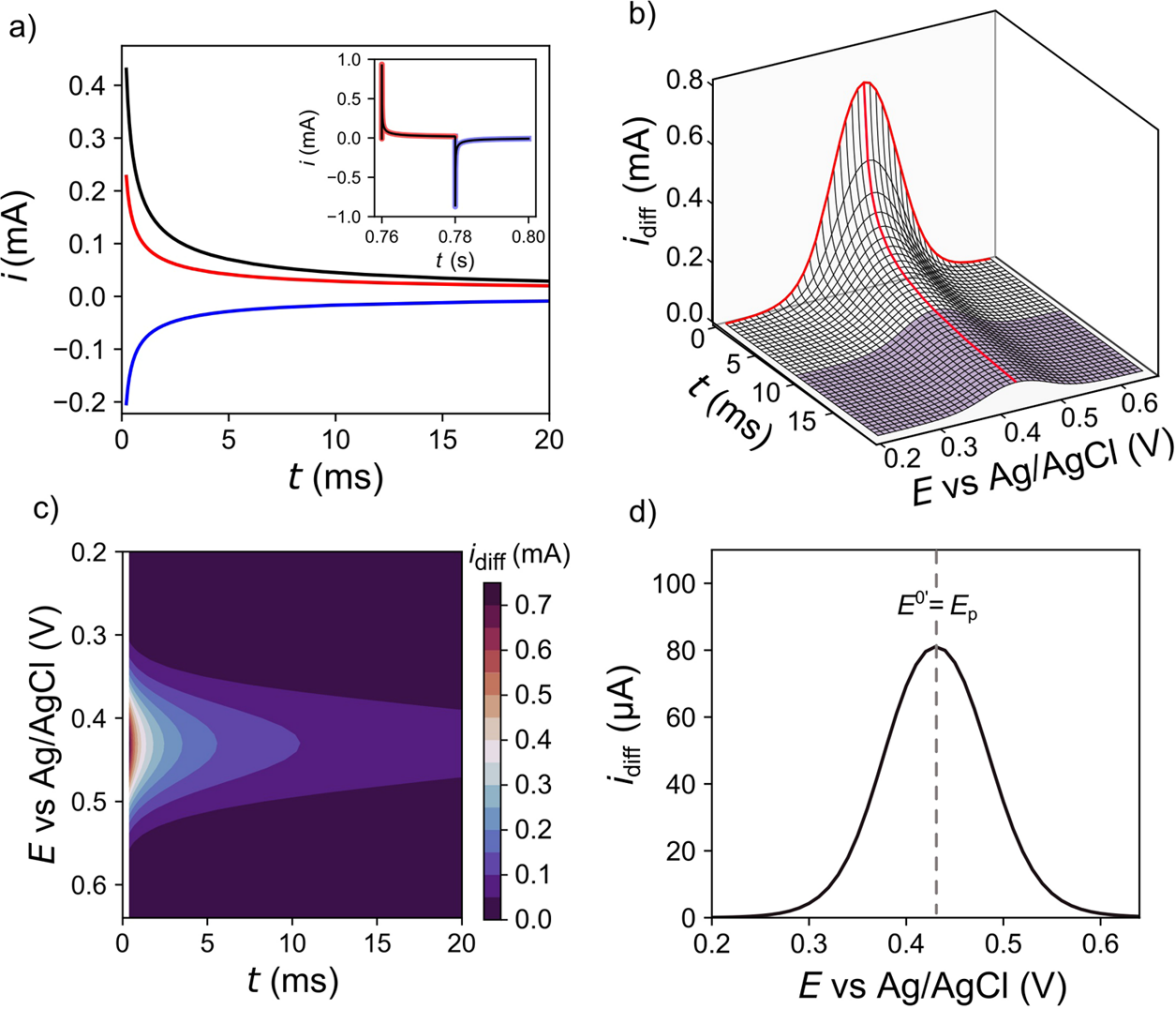
Simulation for an outer-sphere redox couple (a) *i*_for_–*t* (red), *i*_rev_–*t* (blue), and *i*_diff_–*t* for an exemplar SWV pulse cycle. The inset shows the original simulated data used to generate the *i–t* transient data. (b) 3D SWV plot. (c) Color contour plot of *i*_diff_–*t–E*. (d) 2D SWV obtained using a current averaging window of 50–100%. The simulation parameters are Δ*E*_SW_ = 50 mV, *f*_SW_ = 25 Hz, Δ*E*_I_ = 10 mV, *D*_O_ = *D*_R_= 6.7 × 10^–6^ cm^2^/s,^32^*k*^0^ = 5 cm/s,^28^*E*^0^′ = 0.43 V vs Ag/AgCl (3 M KCl), *A* = 0.0707 cm^2^, *T* = 293.15 K.

**Figure 3:**
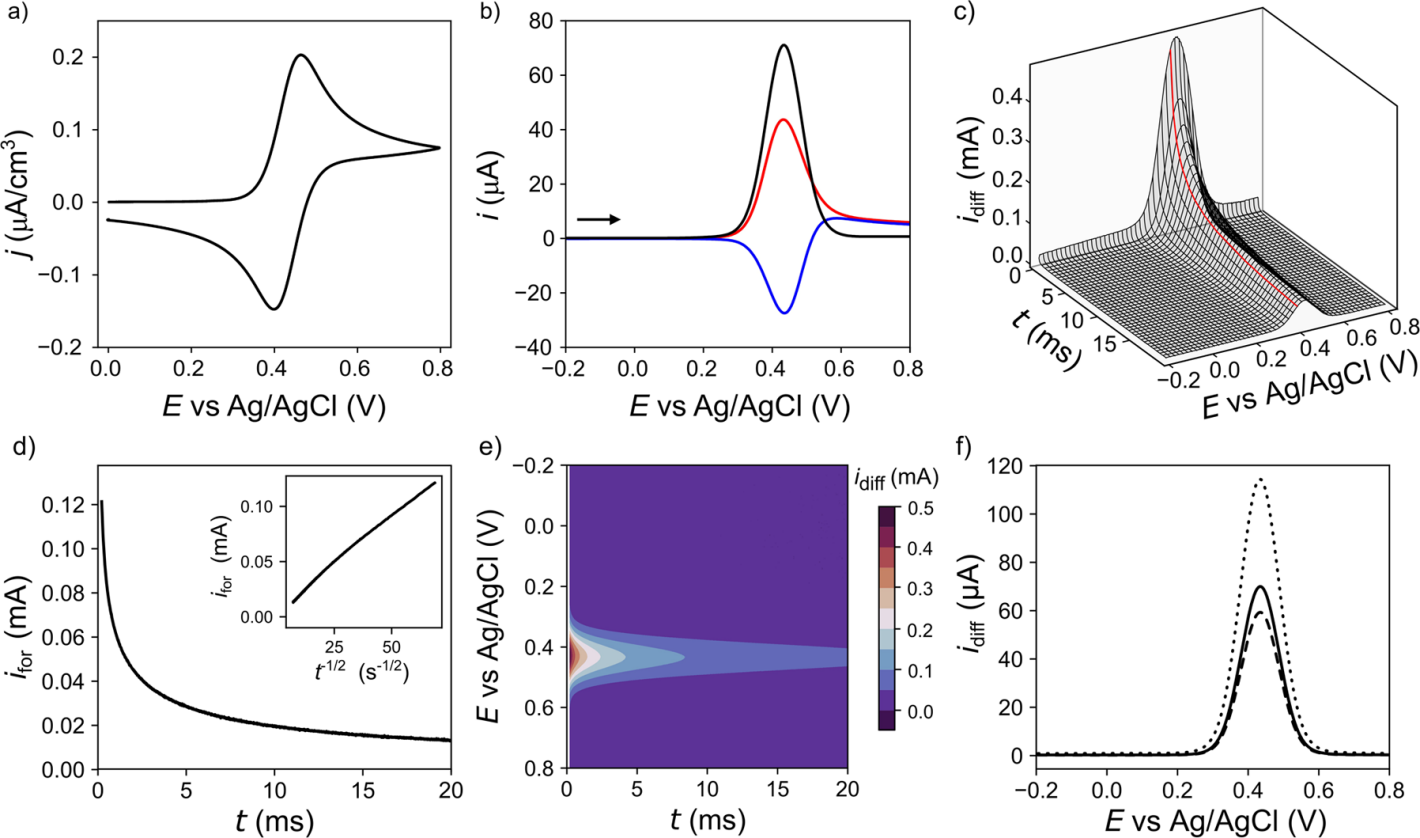
Data for 1 mM FcTMA^+^ in 0.1 M KNO_3_ using a BDD electrode (a) CV response at 0.1 V/s, (b) *i*_for_–*E* (red), *i*_rev_–E (blue), and *i*_diff_–*E* (black), 2D SWV’s (50–100% current averaging). (c) 3D SWV response, (d) *i*_for_–*t* curve at *E* = 0.351 V vs Ag/AgCl with an inset of *i*_for_–*t*^–1/2^, (e) color contour map of *i*_diff_–*t*–*E* with *i*_diff_ as the color scale, (f) 2D *i*_diff_–*E* SWV response with current averaging 50–100% (solid), 1–100% (dotted) and 90–100% (long dashed). SWV parameters: Δ*E*_SW_= 50 mV, *f*_SW_= 25 Hz, Δ*E*_I_= 4 mV, quiet time = 2s, current range 0.1 mA/V.

**Figure 4:**
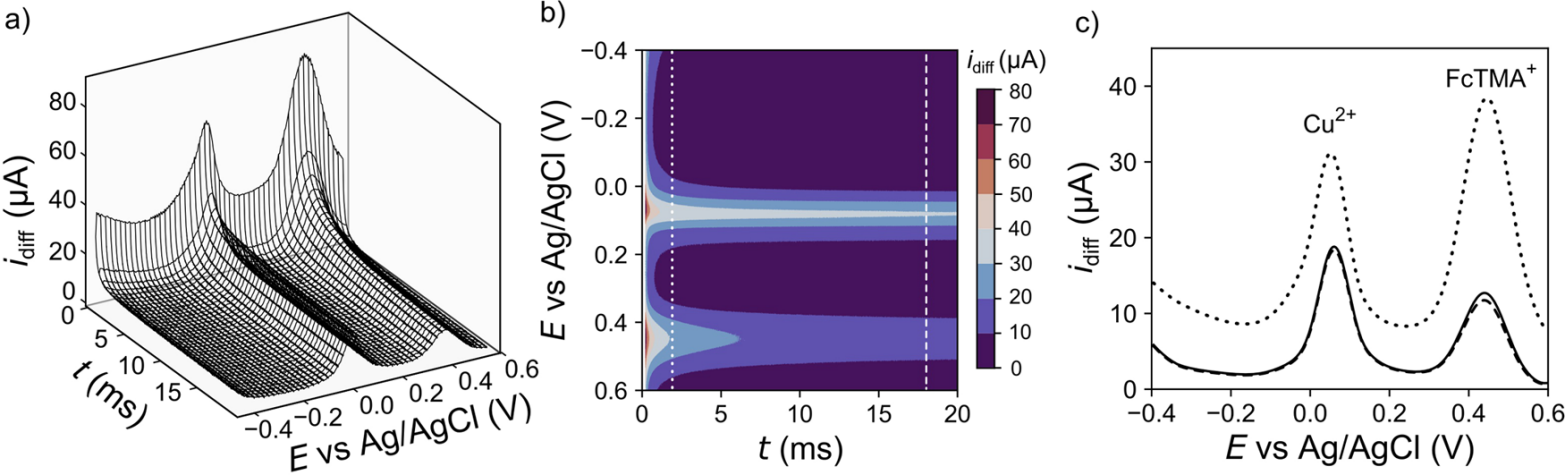
Cu^2+^ (400 μM) and FcTMA^+^ (100 μM) in 0.1 M KNO_3_, pH 5.8 using a BDD electrode. (a) 3D SWV response, (b) color contour map of *i*_diff_–*t*–*E* with *i*_diff_ as the color scale; the dotted line corresponds to 10% (2 ms) of the pulse, while the dashed line corresponds to 90% (18 ms) of the pulse, (c) 2D SWV obtained by current averaging over 1–10% (dotted), 50–100% (solid) and 90–100% (dashed) of the *i*_diff_*-t* transients.

**Figure 5:**
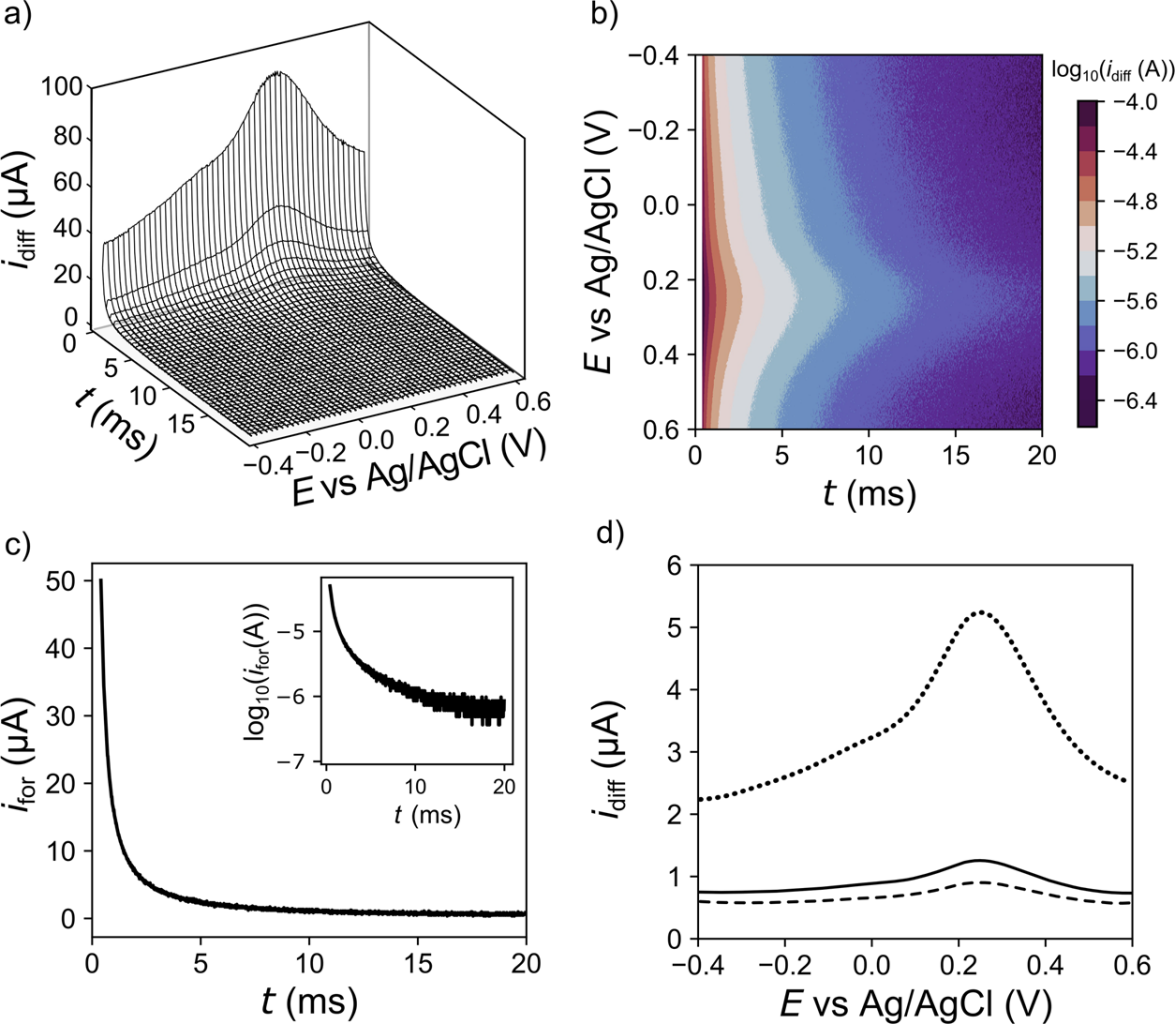
BDD-Q pH electrode in 0.1 M KNO_3_(pH 5.7). (a) Plot of the 3D SWV response, (b) color contour map of *i*_diff_–*t*–*E* with *i*_diff_ as the color scale, (c) *i*_for_–*t* transient at *E* = 0.249 V vs Ag/AgCl with the inset plotted on a log_10_ scale, (d) 2D SWV response after current averaging between 50–100% (solid), 2–100% (dotted), and 90–100% (dashed).

**Figure 6:**
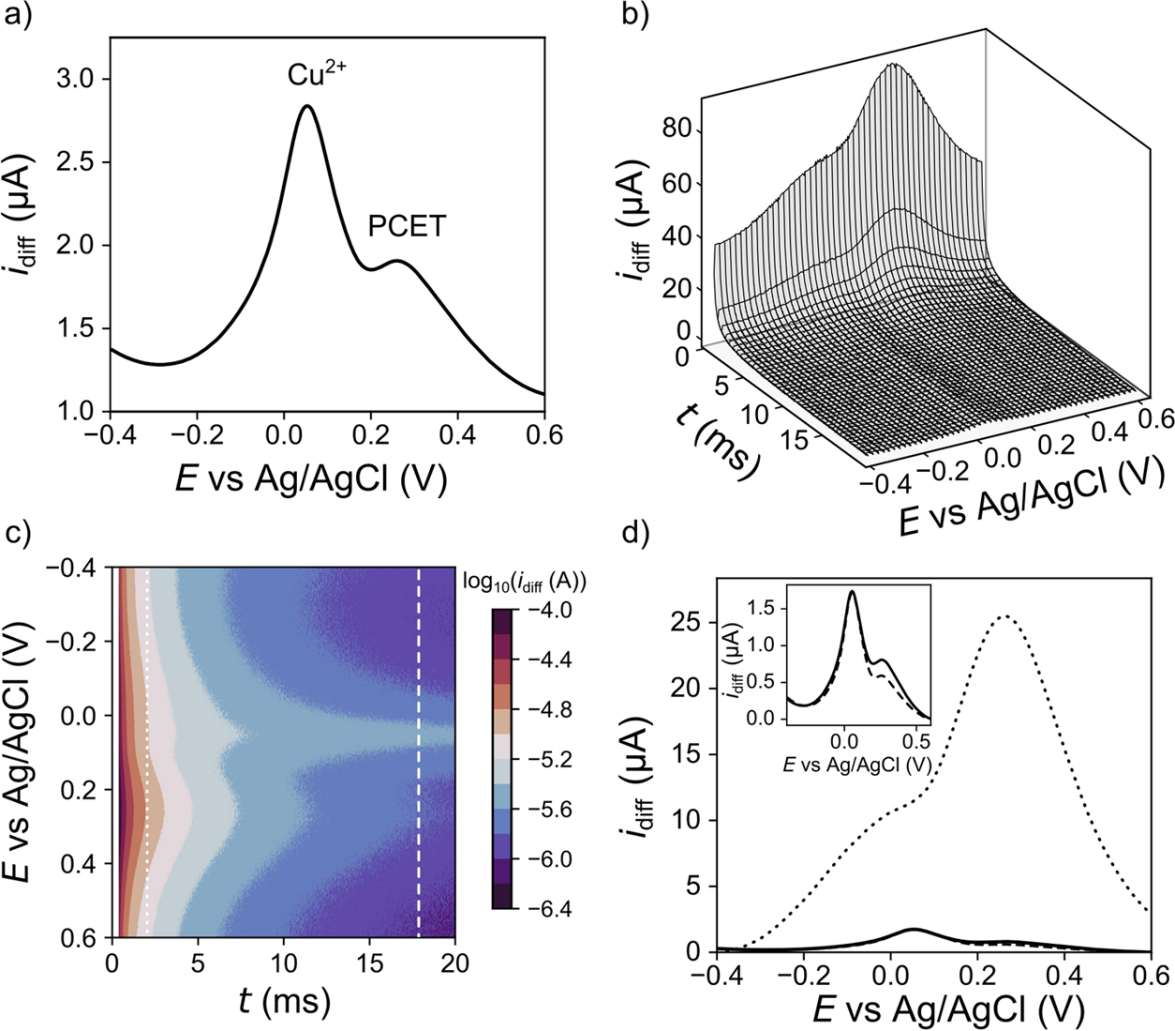
BDD-Q electrode in 0.1 ppm of Cu^2+^ in 0.1 M KNO_3_ (pH 5.3). (a) SWV response using the potentiostat standard 50–100% current averaging window, (b) 3D SWV, (c) color contour map of log_10_(*i*_diff_–*t*–*E*) with *i*_diff_ as the color scale, (d) 2D SWV using current averaged windows of 2–10% (dotted), 90–100% (dashed), and 50–100% (solid), inset: zoomed-in 90–100% and 50–100% 2D SWV results.

For each pulse cycle in the SWV trace, the forward (red line) and reverse (blue line) *i*–*t* trace can be converted into a single *i*_diff_–*t* transient (black line) by calculating (*i*_for_ – *i*_rev_) for each time point, as shown in Figure 2a, using simulated data. Note that this requires the start of the forward and reverse pulses for each cycle to be set to *t* = 0 s. To demonstrate 3D representation, all data shown in Figure 2 is COMSOL simulated for an outer sphere fast electron transfer (reversible) redox couple with only faradaic processes considered.^31^ Simulation details are found in the figure legend and Supporting Information 2. For all data collected (*vide infra*), *f*_SW_ = 25 Hz, which means one pulse cycle lasts 40 ms, thus the forward (red) and reverse (blue) pulses are 20 ms each, which is the time domain over which *i*_diff_ is plotted. The *i*_diff_–*t* transients, when plotted against *E* of the linear staircase ramp (over the range 0.20 to 0.64 vs Ag/AgCl) generate a 3D plot of *i*_diff_–*t–E,* as shown in Figure 2b. To plot Figure 2b on a meaningful scale, the first 0.4 ms are removed, as at very early time points large currents are passed due to the *t*^–1/2^ dependence of the faradaic current. When considering real experimental data later (Figures 3–6), double-layer charging currents are removed, which occur over similar time points. Figure 2b can be viewed in two ways by visualizing either (i) the characteristic *i*_diff_–*E* peak-shaped response at different *t* values (the red line in Figure 2b shows the *i*_diff_–*E* response at *t* = 0 ms). (ii) *i*_diff_–*t* transient response at different *E* values (the red line in Figure 2b shows the *i*_diff_–*t* response at the peak potential, *E*_p_).

The data can also be displayed using a color contour plot of *i*_diff_–*t*–*E*, as shown in Figure 2c, where the highest currents are observed at the shortest times. The 3D SWV measurement can be transformed into a conventional single 2D SWV by applying a current averaging window over a defined period of each forward and reverse pulse to produce a single current value per *E*. For the example shown in Figure 2d, the 2D SWV is generated by averaging over the last 50–100% of the *i*_diff_–*t* transients, for each *E* value (highlighted in purple in Figure 2b). Under reversible electron transfer conditions, *E*_p_ of the 2D SWV response is equal to the formal potential, *E*^0^′.^9^

### Numerical Simulations

Numerical simulations were performed in COMSOL Multiphysics 6.1 (COMSOL AB, Sweden) and are described in detail in Supporting Information 2. The corresponding model report is also provided.

### Data Analysis

Python 3.9 was used to both process the *i–t* transients into 3D SWV plots and apply a current averaging window to generate the 2D SWV (Figures 2–6). While the code, available on GitHub,^33^ is optimized for the data output by the AutoLab potentiostat, it can easily be adapted for other potentiostats. For the voltammograms in Figure 6d to be visible on the same current axis, linear background subtraction was employed for each current averaging window, with the baseline set as the minimum current for each SWV response.

## Results and Discussions

### Outer Sphere Electron Transfer and Metal Electrodeposition/Stripping

To understand how 3D SWV plots can be experimentally exploited, we first consider in more detail a soluble redox couple (R/O) that undergoes one electron, fast electron transfer, where the forward and backward reactions are in equilibrium. The *i–t* response for the oxidation of the initial reduced species (R), following a step in the potential from a value where no current flows to one that is sufficient to drive electron transfer at a diffusion-limited rate, can be described using the Cottrell equation. This equation assumes semi-infinite planar diffusion is the mode of mass transport to/from the electrode and the concentration of R at the electrode surface is zero.^34^ To account for nonzero surface concentrations of R, the Cottrell equation can be modified to include a dependence on the overpotential. Both forms of the Cottrell equation predict a current response with an inverse dependence on *t*^1/2^.^9,12^

The oxidation of FcTMA^+^ on a metallic electrode is considered to be a fast electron transfer process with a *k*^0^ value of 5 ± 2 cm/s.^35^ This agrees with the CV for oxidation of 1 mM FcTMA^+^ in 0.1 M KNO_3_ on Au at 0.1 V/s, as shown in Supporting Information 3, Figure S4a. Over the potential range, 0.0 to 0.8 V vs Ag/AgCl, the CV response has a peak-to-peak separation (Δ*E*_p_) of 59 mV, very close to reversible.^34^ On BDD, the *k*^0^ for the oxidation of FcTMA^+^ is smaller, measured to be approximately 0.1 cm/s,^28^ and the CV in Figure 3a shows a Δ*E*_p_ of 62 mV (at 0.1 V/s). The two CVs have a half-wave potential, *E*_1/2_, of 0.433 and 0.431 V vs Ag/AgCl for Au and BDD, respectively. Figure 3b shows a conventional SWV (black line for *i*_diff_ vs *E*) recorded using the commercial (AutoLab) potentiostat (which averages over 50–100% of each *i–t* transient). The *E*_p_ value of 0.435 V vs Ag/AgCl is extremely close to the *E*_1/2_ value recorded by CV. Supporting Information 3, Figure S5a shows the SWV data for Au where *E*_p_ also is equal to 0.435 V vs Ag/AgCl.^36^ Electron transfer reversibility can also be interfered from the peak separation between the forward (red) and reverse (blue) waves in Figure 3b (and Figure S5), which is 0 for a reversible reaction on the time scale of the measurement.^9^ The peak separation is 0 mV for the Au electrode and 4 mV for the BDD electrode.

When analyzing the 3D SWV data set, it is important to consider the portion of each *i–t* transient, which is associated with nonfaradaic events. The nonfaradaic current decays to <1% of the initial value, at *t* = × 5 the time constant, *R*_u_*C*_dl_, where *R*_u_ is the uncompensated resistance, and *C*_dl_ is the double-layer capacitance.^37^ For all measurements herein, to minimize the influence of the electrode charging process on the faradaic response of interest, the portion of the *i–t* transients that fall within this time domain are removed from the 3D data set. As the time constant will differ depending on the electrode material used, these parameters were measured. The BDD electrode was found to have a lower *C*_dl_ of 3.3 ± 0.3 μF/cm^2^ than Au = 14.6 ± 0.5 μF/cm^2^; however, *R*_u_ for BDD was slightly higher, i.e., 141 ± 3 Ω vs 131 ± 3 Ω for Au, Supporting Information 4. Overall, the nonfaradaic charging current for the BDD electrode (5*R*_u_*C*_dl_ = 0.163 ± 0.012 ms) decays faster than Au (5*R*_u_*C*_dl_ = 0.300 ± 0.009 ms). Consequently, when using the BDD electrode to monitor faradaic processes, earlier time points on the *i*_diff_*–t* transient can be analyzed. BDD was therefore used as an electrode material for all experiments herein.

Figure 3c shows the 3D SWV data where the first 0.2 ms (1%) has been removed from all *i–t* transients, to account for the nonfaradaic charging current, and the time reset to 0 ms per transient. In Figure 3c as the time increases from 0 to 20 ms, the peak *i*_diff_–*E* response decreases in magnitude, reflecting the decrease in the flux of FcTMA^+^ to the electrode surface. Taking cross sections of the 3D SWV data along the time axis generates 2D SWVs (*i.e. i*_diff_–*E* plots) at discrete time points (i.e., without current averaging), similar to the continuous SWV measurements presented by Abeykoon and White.^22^ Conversely, taking cross sections along the *E*-axis generates *i*_diff_–*t* plots at discrete potentials. An example of the *i–t* transient for the forward pulse (prior to subtraction of *i*_rev_ to obtain *i*_diff_) at *E* = 0.351 V vs Ag/AgCl is shown in Figure 3d. When *i* is plotted against *t*^*–*1/2^ in the inset of Figure 3d, the trace displays an approximately straight line showing the current transient has a *t*^*–*1/2^ time dependence.

As highlighted in Figure 2c, the 3D SWV can also be plotted as a color contour map with *i*_diff_ as the color scale, here each band of color represents 50 μA, as shown in Figure 3e. The rate of the current decay, characterized by the change in the magnitude of the spacing between the different color bands, varies most dramatically at the start of the pulse, due to the steep concentration gradient of FcTMA^+^ at the electrode surface at short time scales. However, there is still a visible change in the signal in the last half of the pulse. As well as plotting *i*_diff_–*E* data for discrete time points, different current averaging windows can also be applied to the 3D SWV data in Figure 3c. Figure 3f shows the resulting 2D SWVs obtained by current averaging from 50–100% (solid black line data), 1–100% (dotted line), and 90–100% (dashed line) of the *i*_diff_*–t* transients. As the start of the current averaging window is moved closer to the start of the pulse, where the current has had less time to decay, the current passed increases. Note, averaging over the whole *i*_diff_*–t* transient (0–100%) has been used as a means of approximating analogue linear scan CV behavior.^38,39^ In Figure 3f, the observed *E*_p_ sits at 0.433 vs Ag/AgCl and is consistent between the three current averaging windows.

In practice, many redox couples of interest do not exhibit fast electron transfer, nor are they categorized as outer sphere. Under these conditions, the *i*_diff_*–t* transients will show a different time dependence to redox couples that exhibit reversible outer sphere electron transfer.^34^ Thus, how the 2D SWV changes in response to the current averaging window utilized will differ for different classes of redox couples. To probe this concept further, we examine the oxidation of FcTMA^+^ and Cu^2+^ reduction on BDD. Metal reduction on BDD is chosen as an example redox process as BDD is used extensively for heavy metal detection in electroanalytical applications due to its large cathodic aqueous potential window.^40,41^ Cu^2+^ reduction to Cu, thermodynamically has one of the more positive metal reduction potentials (*E*^0^ = 0.137 V vs Ag/AgCl)^44^ and is thus more likely to act as an interferent when using voltammetry to detect analytes in real solutions. Below ∼pH 6, Cu^2+^ is the dominant copper species in the potential window 0.6 V to −0.4 V vs Ag/AgCl.^42^Supporting Information 5, Figure S10a shows the first scan CV recorded with a 3 mm diameter BDD disk electrode at 0.1 V/s in a deaerated solution containing 0.1 ppm (1.57 μM) Cu^2+^ in 0.1 M KNO_3_ (pH = 5.3).^40^ The cathodic peak at ∼ – 0.15 V vs Ag/AgCl is due to Cu^2+^_(aq)_ reduction to Cu_(s)_. An appreciable overpotential is required for this process, a consequence of Cu electrodeposition on a non-Cu substrate.^41^ The concentration employed here is in line with the data reported in Figure 6. At ∼ 0.07 V vs Ag/AgCl, an anodic peak is observed due to oxidation (dissolution) of the electrodeposited Cu. The impact of oxygen can be seen in Supporting Information 5, Figure S10b, where the solution is deliberately left aerated.

Depending on whether the SWV potential is scanned reductively or oxidatively will determine whether *i*_for_ is representative of Cu^2+^ reduction or Cu dissolution (stripping). Description of the *i*–*t* transients for metal deposition/stripping on a low energy carbon surface will depend on many factors, which reflect the mechanism of the process. These include, for example, the energetics of surface interactions between metal atoms and the heterogeneous BDD substrate and the mechanism of metal nanoparticle growth/dissolution.^43,44^ Hence, predicting the *i*–*t* transient behavior (*i*_for_ and *i*_rev_) is challenging. It is simpler to experimentally capture the data and use 3D SWV plots to inform the best operational conditions, depending on the objective of the experiment.

Figure 4a,b shows the 3D SWV and 2D color contour maps, respectively, for a solution containing 100 μM FcTMA^+^ (*E*_p_ ≈ 0.441 V vs Ag/AgCl) and 400 μM Cu^2+^ (*E*_p_ ≈ 0.061 V vs Ag/AgCl) in 0.1 M KNO_3_ at pH 5.8. The solution is left deliberately aerated to reflect applications where deaeration of the solution is not possible, e.g., in situ monitoring. The potential was scanned oxidatively from −0.4 to 0.6 V vs Ag/AgCl, hence *i*_for_ represents a stripping current for Cu^2+^/Cu and oxidation of FcTMA^+^, as depicted in Figure S11a (which also shows the *i*_rev_ currents associated with Cu^2+^/FcTMA^2+^ reduction). As highlighted in Figure 4a,b, the FcTMA^+^*i*_diff_ currents decrease more rapidly with *t*, than the Cu^2+^. For example, the FcTMA^+^*i*_diff_ current at *E*_p_ decreases from 27 to 12 μA from 0.2–20 ms, while for Cu^2+^, the *i*_diff_ current (at *E*_p_) decreases only from 26 to 20 μA, over the same time period.

Given the different *i*_diff_–*t*–*E* responses for the two redox couples, the current window over which *i*_diff_ is averaged will play a significant role in controlling the relative magnitudes of the 2D SWV currents associated with the two redox species. Figure 4c shows the 2D SWVs for current–time averaging windows of 1–10% (dotted), 90–100% (dashed), and the potentiostat standard 50–100% (solid). When 1–10% is selected, the FcTMA^+^ signal is dominant over the Cu^2+^ signal; however, this behavior is reversed when analyzing over 50–100% and 90–100% of the window. This data highlights that for redox couples that exhibit different electron transfer kinetics/reaction mechanisms, the current–time sampling window will control the relative heights of the 2D SWV currents in different ways. This concept is explored further as a means to reduce the voltammetric impact of interfering species in relation to a target analyte.

### Voltammetric pH Sensing

Surface-bound redox couples have been widely used for studying the fundamentals of electron transfer kinetics and in applications such as electrochemical sensing.^45,46^ An example of one such sensor system is the BDD-Q pH electrode, which utilizes surface-integrated quinones to voltammetrically measure solution pH via proton-coupled electron transfer (PCET).^5,47^ Such electrodes provide a Nernstian pH response and excellent pH accuracy in unbuffered solutions. The quinones are produced using a laser to directly convert regions of the BDD surface to sp^2^ carbon (BDD-Q laser pattern shown in Figure S1, Supporting Information 1).

A key challenge when moving to real-world matrices such as river and tap (drinking) water is the presence of redox-active interferents, which, if they have redox potentials in the region of interest, will convolute with the quinone signal.^5^ Cu^2+^ reduction/dissolution is the most problematic heavy metal, as the *E*^0^′ overlaps with the BDD-Q voltammetric signature in the pH region 5–7 (∼0.1 to 0.25 V vs Ag/AgCl^43^). Cu^2+^ concentrations typically range between 0.0001 and 1 ppm (mg/L) in groundwater and 0.02 and 133 ppm in UK river water.^48,49^ To mitigate against this interference, we look to understand how the *i*_diff_–*t*–*E* response of the two redox systems differs and how that difference can be used advantageously in SWV sampling.

Redox couples bound to an electrode surface have no dependence on the mass transport of the redox species. The rate of electron transfer associated with the finite number of redox groups controls the *i*–*t* transients with an exponential dependence on *t*.^34,50^ The quinone groups of the BDD-Q pH electrode add an extra degree of complexity as they undergo concerted 2H^+^/2e^–^ PCET.^47,51,52^ They are also present at submonolayer surface coverage (∼10^–11^ to 10^–12^ mol/cm^2^),^47^ resulting in small quinone CV currents on top of the background charging signal, Figure S12, Supporting Information 6, necessitating the use of SWV. While in the literature (and to the best of our knowledge) the *i*–*t* behavior of a 2H^+^/2e^–^ proton PCET reaction has not been previously reported, here we examine its experimental form to investigate whether it also follows an exponential time dependence. As the BDD-Q electrode has a larger background charging current (5*R*_u_*C*_dl_ = 0.371 ± 0.014 ms, see Supporting Information 4, Figure S9) than a BDD electrode due to the presence of sp^2^ carbon, the first 2% of the *i*–*t* response is removed.

Figure 5a shows the 3D SWV data for a BDD-Q electrode in 0.1 M KNO_3_ for the same SWV parameters used previously in Figures 3 and 4, over a potential range of −0.4 to 0.6 V vs Ag/AgCl. While the quinone *i*_diff_ signal (peaking at ∼0.25 V vs Ag/AgCl) can be seen at early time points between 0.4 and 2 ms (2–10%), it is not easily visible on this *z*-axis scale after 5 ms (25–100% of the *i*_diff_ transient). This is rectified by plotting the current as a color contour map, on a log_10_ current scale, Figure 5b. The *i*–*t* transient (for *i*_for_–*t* rather than the combination current *i*_diff_) is shown at *E*_p_ = 0.249 V vs Ag/AgCl, Figure 5c. *i*_for_ decays by an order of magnitude within the first 2 ms and continues to decay until ∼5 ms, after which between 5 and 20 ms, it is difficult to distinguish the current from the digital signal limit of the potentiostat current resolution. When the current is plotted on a log_10_ scale, in the inset of Figure 5c, over ∼0.4–2 ms, the response is linear, suggesting an exponential dependence, after which it is hard to differentiate between the quinone signal and background noise. For the 2D *i*_diff_–*E* SWV’s shown in Figure 5d, the largest signal is seen when averaging over 2–100% of the *i*–*t* transients (dotted line), which decreases upon going to 50–100% (solid line) and further still using 90–100% (dashed line).

To examine how the Cu^2+^ signal interferes with the BDD-Q pH response, a concentration on the higher end of the range of 0.1 ppm (1.6 μM) was used where the Cu^2+^ current signals will be significant. Interference of the Cu^2+^ signal with the PCET quinone response can be seen in Figure 6a when using the potentiostat standard current averaging window (50–100%) to generate the 2D SWV in a solution of 0.1 M KNO_3_ (pH 5.3). The Cu^2+^ SWV signal (*E*_p_ = 0.065 V vs Ag/AgCl) dominates the voltammetric response, with the shoulder on the right-hand side corresponding to the quinone signal.

The 3D SWV data in Figure 6b, however, shows that at the early time points ∼0.4–2 ms (2*–*10%), the quinone PCET response dominates the signal, with the Cu^2+^ response barely distinguishable. The quinone current response rapidly decays by an order of magnitude over 5 ms. The Cu^2+^ behavior is better discerned when the current is plotted as a contour plot on a log_10_ scale, Figure 6c. The Cu^2+^ signal decays at a slower rate and dominates at times >5 ms (>50%), which is the region normally sampled by a commercial potentiostat. Figure 6d shows the 2D SWV data recorded using the standard potentiostat current averaging window of 50*–*100% (solid line), 90*–*100% (dashed), and 2*–*10% (dotted). For these responses to be visible on the same *y*-axis, linear background subtraction was employed (see Experimental Section). As Figure 6d shows, the quinone response is the dominant signal when averaging over 2*–*10% of the pulse; the shoulder to the left-hand side of the quinone peak is the Cu^2+^ signal. In contrast, the Cu^2+^ signal is enhanced by using a later current averaging window, 90*–*100% of the pulse. Hence, by probing the start and end of the *i*_diff_–*t* transients separately in one measurement, it is possible not only to deconvolute the quinone signal from that of the Cu^2+^ but also to resolve the interferent signal. Ultimately, it would also be possible to quantify the Cu^2+^ concentration from this signal (subject to calibration experiments).

## Conclusions

For practical electroanalytical applications, target analytes are rarely found in isolation; often other redox active species are present, which may convolute with the analyte. SWV is commonly used in electroanalysis to enhance analyte signals. By capturing all the *i*–*t* data associated with a SWV, we have demonstrated how it is possible, through judicious choice of the current averaging time window, to enhance the analyte signal while simultaneously minimizing that due to the redox interferent. This is possible when the analyte and interferent redox species show appreciably different SWV *i*–*t* behavior.

We illustrate this through consideration of a surface-confined PCET process at a BDD-Q electrode (used in voltammetric pH sensing) versus Cu^2+^ electrodeposition/stripping. The PCET *i*–*t* responses are exponential in shape, while those associated with Cu^2+^/Cu decay much slower (a more complex process). 3D experimental *i*–*t*–*E* plots were employed to visualize such differences and were shown to be especially useful for processes difficult to analytically define. By using a current window, which averages over earlier time points (2–10%), it was possible to enhance the PCET (pH) signal and reduce that associated with Cu^2+^/Cu. Conversely, averaging over later time points (≥50–100%) enhanced the Cu^2+^/Cu signal compared to that for pH. Movement of the current–time averaging window is similar to changing *f*_SW_ in SWV; however, the former can be done using one measurement, whereas the latter requires sequential changes to *f*_SW_ and is thus much more time-consuming. Such understanding can also be used with SWV at sp^2^-bonded carbon electrodes, such as screen-printed electrodes, to reduce the background PCET signals associated with surface-confined quinones^53^ and discriminate against oxygen interferences. Finally, analysis of the full *i*–*t* response can be used to optimize not only SWV but also other pulsed voltammetric techniques.

## Data Availability

All raw data used in the preparation of this manuscript can be found at the Warwick Research Archive Portal http://wrap.warwick.ac.uk/185218. These data are provided free of charge.

## Supporting Information Available

BDD-Q electrode patterns; COMSOL simulations; additional voltammetry of FcTMA^+^; extracting the uncompensated resistance and double layer capacitance; additional voltammetry of Cu^2+^; CV response of BDD-Q electrode (PDF)
A separate COMSOL model (PDF)
