# Supplementary material for: A Current Averaging Strategy for Maximizing Analyte and Minimizing Redox Interference Signals with Square Wave Voltammetry
Source: Anal Chem. 2024 May 26;96(23):9561–9. doi: 10.1021/acs.analchem.4c01053 (PMC11170553; doi:10.1021/acs.analchem.4c01053)
Supplement: Supplementary file 1 — ac4c01053_si_001.pdf [file ac4c01053_si_001.pdf]

Supporting Information

A Current Averaging Strategy for Maximizing Analyte and Minimising Redox Interference Signals with Square Wave Voltammetry

Katherine J. Levey and Julie V. Macpherson*

Department of Chemistry, University of Warwick, Coventry, CV4 7AL, UK

*j.macpherson@warwick.ac.uk

Contents

SI.1. BDD-Q Electrode Patterns S2

SI.2. COMSOL simulations S3

SI.3. Additional Voltammetry of FcTMA^{+} S6

SI. 4. Extracting the Uncompensated Resistance and Double Layer Capacitance S7

SI 5. Additional Voltammetry of Cu^{2+} S11

SI 6. CV response of BDD-Q Electrode S13

SI 7. References S13

SI.1. BDD-Q Electrode Patterns

A pattern of 24 concentric rings, each with a thickness of 15 µm and a spacing of 50 µm between each ring, was laser machined into the BDD-Q electrode (d = 3 mm), Figure S1. The laser pulse spot size was ~ 6 µm in diameter. As such each of the 15 µm diameter concentric rings are made up of five overlapping rings (lasered sequentially), with a pitch of 3 µm between the centre of each overlapping ring. Each laser pulse is spaced 1.5 µm apart.

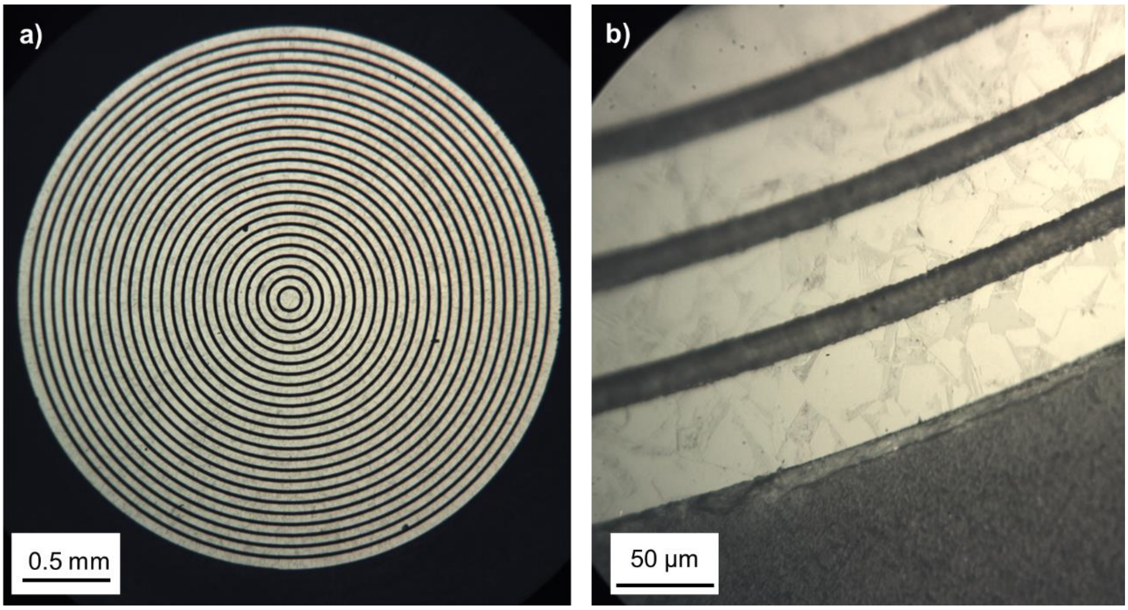

Figure S1. Optical microscope images of the BDD-Q electrode (a) the whole electrode and (b) a magnified section near the edge of the BDD disk.

SI.2. COMSOL simulations

Numerical simulations were formulated using COMSOL Multiphysics 6.1™ (COMSOL AB, Sweden) to model a one-electron, fast electron transfer, outer sphere redox reaction. The mass transport of species O (oxidised) and R (reduced) is defined by solving the Nernst-Planck partial differential equations using the transport of dilute species node in COMSOL.[1] The generalised design described by Equation S1 assumes that diffusion is the only mode of mass transport and that the electric double layer has no effects on the faradaic process. Therefore, electroneutrality is assumed throughout the solution and convection can be omitted as the solution is unstirred.

$$J_i = -\left(D_i \frac{\partial C_i}{\partial x} \right) \tag{S1}$$

D_i and C_i are the diffusion coefficient and concentration of species i and J_i is the flux normal to the electrode surface.

The rate of electron transfer at the electrode surface is defined by the rate equation described by Equation S2 in terms of O and R.

$$J_\mathrm{R} = -J_\mathrm{O} = k_\mathrm{b} C_\mathrm{R} - k_\mathrm{f} C_\mathrm{O} \tag{S2}$$

Equation S2 also includes the conservation of mass, as the rate of the reduction reaction must be equal and opposite to that of the oxidation. k_f is the forward rate constant for reduction and k_b is the backward rate constant for oxidation as defined by Butler-Volmer kinetics in Equation S3 and S4.

$$k_f = k^0 \exp\left(-\frac{\alpha F}{RT}(E - E^{0'}) \right) \tag{S3}$$

$$k_b = k^0 \exp\left(\frac{(1-\alpha)F}{RT}(E - E^{0'}) \right) \tag{S4}$$

k^0 is the heterogeneous electron transfer rate constant (cm/s), where α is the transfer coefficient (assumed to be 0.5). R, T and F are the ideal gas constant (J/K mol), temperature (K), and Faraday's constant (C/mol) respectively. Full details of the computational model can be found in the additional COMSOL model report provided.

Geometry and Mesh

The Nernst-Planck equation was solved for the one-dimensional model shown in Figure S2, to reduce computation time, but the model could be adapted to other geometries. Boundary point 1 (BP1) corresponds to the electrode surface (x = 0) and BP2 to the position of the reference/counter electrode (x = 2.5 mm, also equal to the total width of the cell model, L). There are ~250 mesh elements, with the finest elements being close to the electrode surface at a size of 1 nm, growing at 5% per element. The mesh grows coarser with the maximum element size being 1/50th of the total width of the model (L = 2.5 mm). The equations were discretised using linear Lagrange elements.

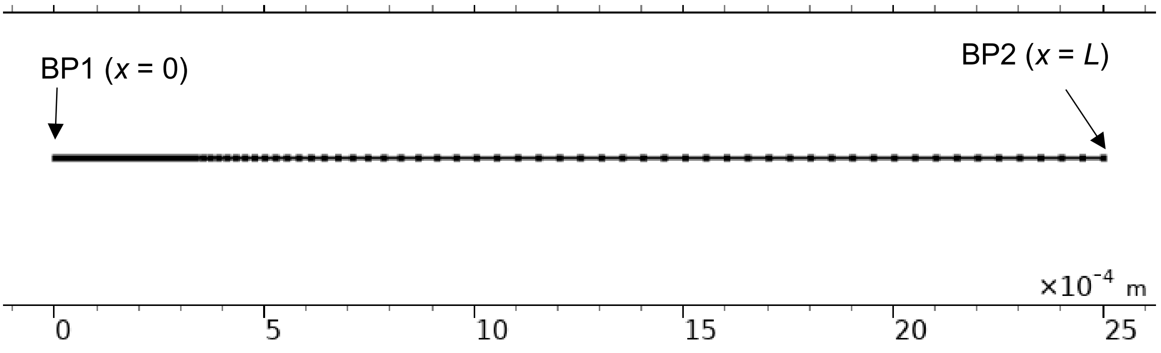

Figure S2. Geometry and mesh used for the finite-element simulations across the whole model L = 2.5 mm and the domain corresponds to the solution phase.

Initial and Boundary Conditions

The initial concentrations (t = 0 s) of each species were set across the domain to $C_R(x, 0) = 1$ mM and $C_O(x, 0) = 0$ mM and fixed at bulk to $C_R(L, t) = 1$ mM and $C_O(L, t) = 0$ mM. The waveform, $E(t)$, was applied in the COMSOL simulation using a piecewise function of the SW waveform shown in Figure S3 matching the following parameters $\Delta E_{SW} = 50$ mV, $f_{SW} = 25$ Hz $\Delta E_I = 10$ mV.

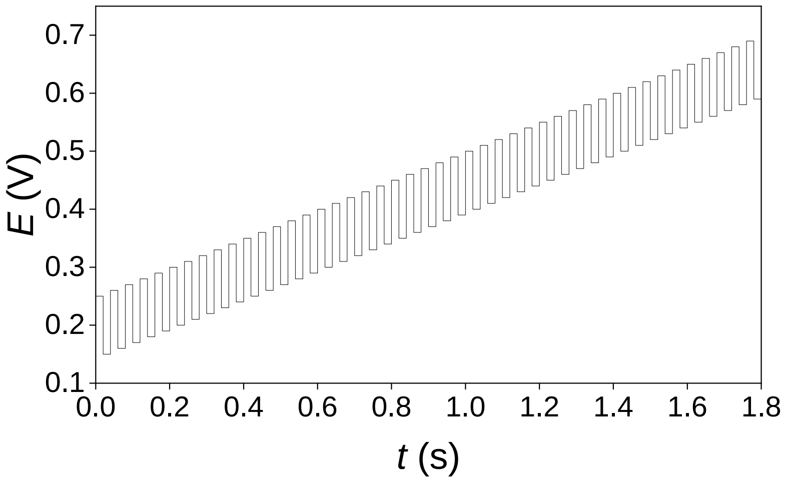

Figure S3. Potential step waveform for the SWV parameters: ΔE_{SW} = 50 mV, f_{SW} = 25 Hz ΔE_{I} = 10 mV.

Computational Simulations and Hardware

Due to the sharp potential step edges used, when the potential is switched, the sudden change can lead to large overshoots in the current. Therefore, an explicit event was set at the start of each potential step edge to reinitialise the solver. In total, the simulation took ~ 50 minutes to solve using the same 10 µs (10 kHz) sampling time employed experimentally. Numerical simulations were formulated using COMSOL Multiphysics 6.1™ (COMSOL, Sweden) using the Chemical Reaction Engineering Module. The simulations were run on HP EliteDesk 800 desktop computer equipped with 64 GB RAM.

SI.3. Additional Voltammetry of FcTMA$^+$

The CV response for 1 mM FcTMA$^+$ in 0.1 M KNO_3 recorded at 0.1 V/s, is shown in Figure S4 on (a) Au and (b) BDD disk electrodes. The key difference is the background capacitive current which is larger on the Au electrode (Figure S4a) than on the BDD electrode (Figure S4b).

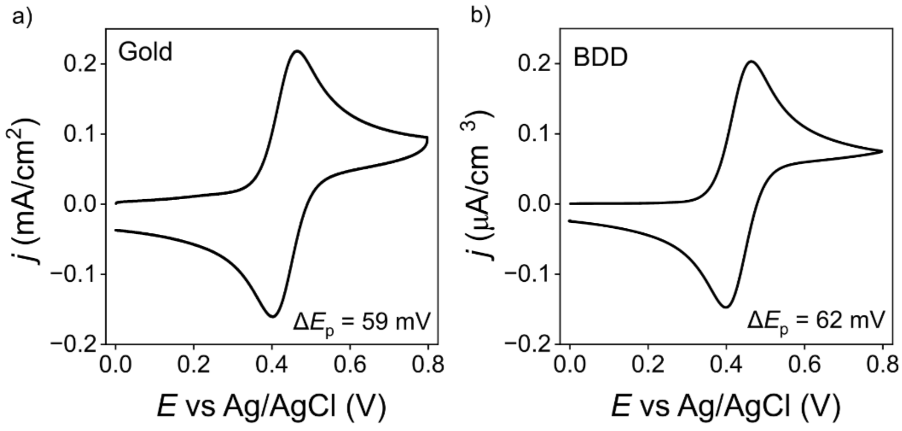

Figure S4. CVs of 1 mM FcTMA$^+$ in 0.1 M KNO_3 on (a) Au (purged with N_2) (d = 2 mm) and (b) BDD (d = 3 mm) at 0.1 V/s.

When comparing the forward and reverse SWV response for the Au and BDD electrodes, for ΔE_{SW} = 50 mV, f_{SW} = 25 Hz, ΔE_I = 4 mV and a quiet time of 2 s, the responses are very similar. There is 0 mV separation between the forward and reverse peaks (E_p) for the Au electrode, and 4 mV (equivalent to the linear ramp step size) for the BDD electrode.

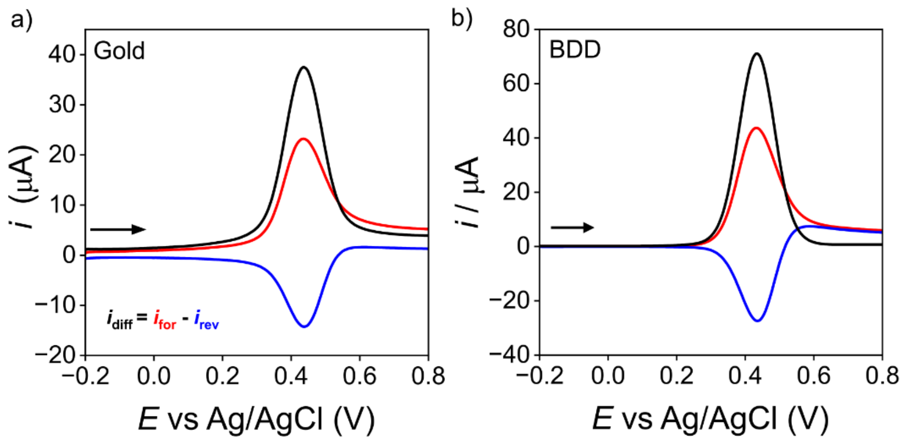

Figure S5. SWV of i_{for} (red), i_{rev} (blue) and i_{diff} (black) versus E (*vs* Ag/AgCl) for 1 mM FcTMA$^+$ in 0.1 M KNO_3 on (a) Au and (b) BDD. SWV parameters: ΔE_{SW} = 50 mV, ΔE_I = 4 mV, f_{SW} = 25Hz.

SI. 4. Extracting the Uncompensated Resistance and Double Layer Capacitance

The RC time constant was estimated across the potential range of interest *e.g.* −0.2 to 0.8 V vs Ag/AgCl by collecting *i-t* data in 0.1 M KNO_3 at 250 Hz (τ = 4 ms) instead of 25 Hz (τ = 40 ms) due to the fast *i-t* decay expected at this solution conductivity (~ 12.8 mS/cm). The sampling time of the current was reduced for this measurement by an order of magnitude from 10 µs to 1 µs to maximise the number of data points. The *i-t* transients for n = 502 steps were fitted using Equation S5,[2] where ΔE is the height of the potential step, R_u is the uncompensated resistance and C_{dl} is the electrochemical double-layer charging capacitance.

$$i(t) = \frac{\Delta E}{R_u} \exp\left(-\frac{t}{R_u C_{dl}}\right) \quad \text{(S5)}$$

Note, the potentiostat does not instantaneously change to apply the exact input potential at the working electrode when the potential is switched, this corresponds to the bandwidth setting used for the control loop. A bandwidth of 1 MHz was employed (ultra-high speed) and the first 5 µs are not included to account for the time taken to switch the potential.

An example of two (of the 502) *i-t* transients recorded in 0.1 M KNO_3 with the BDD electrode and fit using Equation S5, are shown in Figure S6. The raw data is shown using black circles and the red lines show the fit using Equation S5. Also given are the extracted R_u and C_{dl} values.

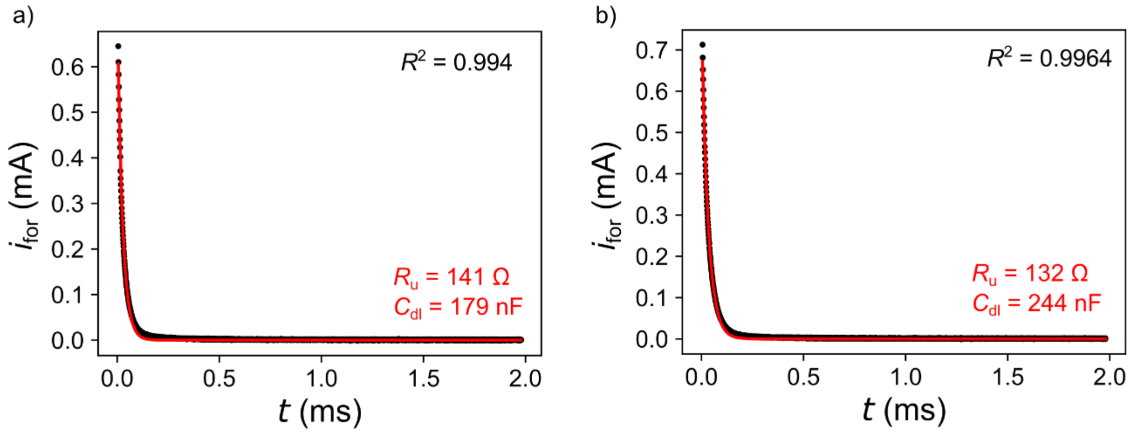

Figure S6. Examples of i_{for} - t transients recorded using a BDD electrode (d = 3 mm) and fit employing Equation S5 (a) at E = −0.301 V (stepping the potential from −0.355 to −0.251 V) and (b) at E = 0.501 V (stepping the potential from 0.447 to 0.551 V).

The C_{dl} and R_u values calculated by fitting the i_{for} and i_{rev} transients (n = 502) over the potential range, −0.2 to 0.8 V are shown in Figure S7. The average C_{dl} (Figure S7a) was 231 ± 19 nF (3.3 µF/cm^2) and the average R_u (Figure S7b) was 141 ± 3 Ω. Note the first full cycle of the

SWV is not included in the calculated C_{dl} and R_u values as the first potential step in the waveform is only ΔE_{SW} instead of 2 ΔE_{SW}, Figure S3. It is assumed that at $t = 5R_uC_{dl}$, 99% of the charging current has decayed. For the BDD electrode across the potential range of interest, an average $5R_uC_{dl}$ value of 0.163 ± 0.012 ms was measured. Therefore, the first 1% of the *i-t* transients (i_{for}, i_{rev} and i_{diff}) was excluded when generating the 3D SWV. All fits have R^2 values above 0.99.

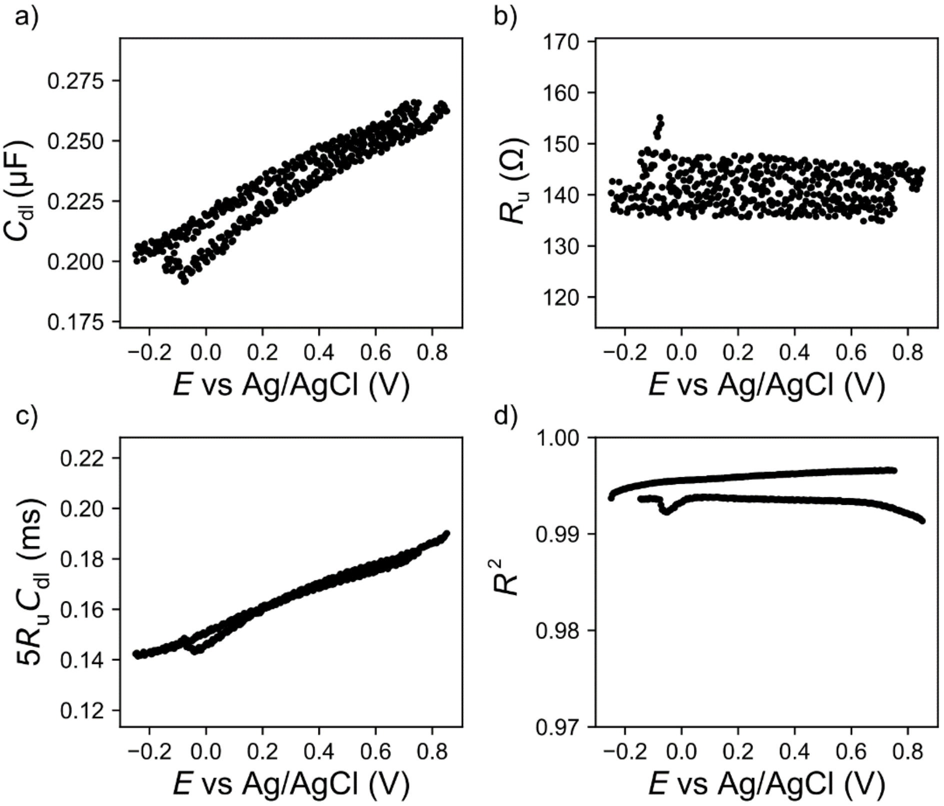

Figure S7. For the potential window used in Figure 3 using a BDD electrode in 0.1 M KNO_3 at 250 Hz the calculated (a) C_{dl}, (b) R_u, (c) ×$5R_uC_{dl}$ and (d) the corresponding R^2 values to the fits using Equation S5. The parameters used for the SWV are the same as Figure S5.

For the Au electrode, the average C_{dl} (Figure S8a) was 459 ± 15 nF (14.6 µF/cm^2) and the average R_u (Figure S8b) was 131 ± 3 Ω. An average $5R_uC_{dl}$ = 0.300 ± 0.009 ms was measured. All fits have R^2 values above 0.98 as can be seen in Figure S8d.

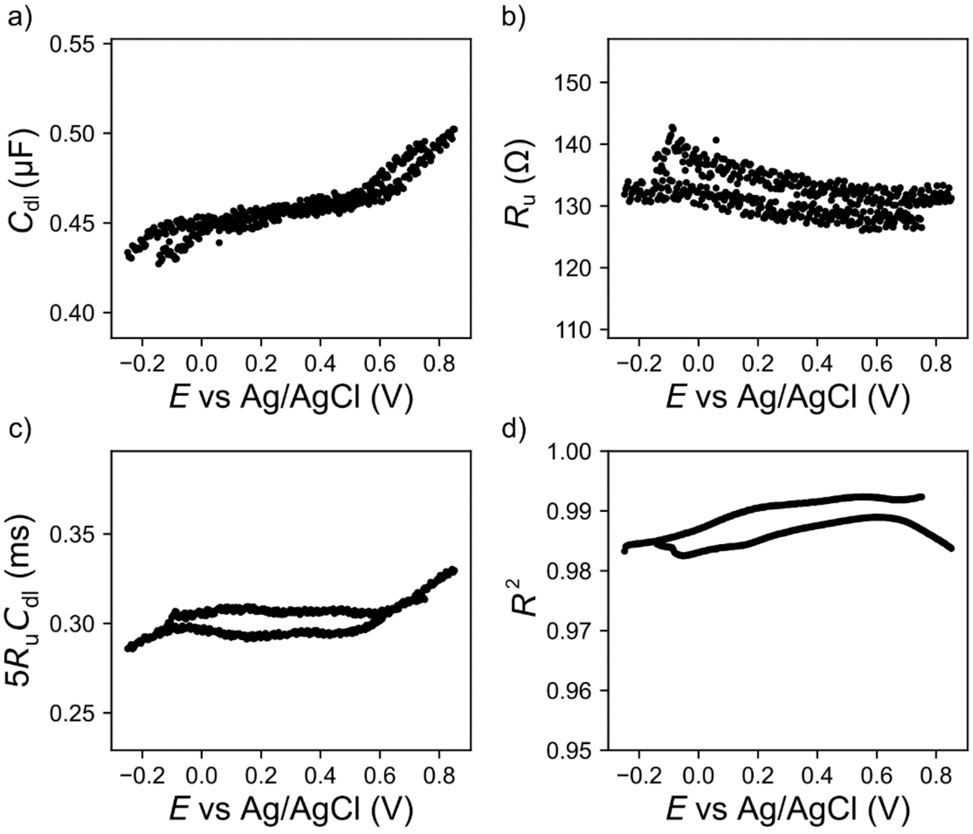

Figure S8. For the potential window used in Figure 3 with an Au electrode (d = 2 mm) in 0.1 M KNO_3 at 250 Hz the calculated (a) C_{dl}, (b) R_u, (c) $\times 5R_uC_{dl}$ and (d) the corresponding R^2 values to the fits using Equation S5. The solution was purged with N_2 before the measurement and all other parameters used for the SWV are the same as Figure S5.

For the BDD-Q electrode, the region between −0.6 V and −0.2 V *vs* Ag/AgCl is used to estimate the $R_u\,C_{dl}$ values as there is no discernible voltammetric signal from the quinone PCET. The average C_{dl} (Figure S9a) was 470 ± 15 nF (6.7 µF/cm^2) in agreement with previous work,[3] and the average R_u (Figure S9b) was 158 ± 3 Ω. The average $5R_uC_{dl}$ = 0.371 ± 0.014 ms calculated from the data shown in Figure S9b. Therefore the first 2% of all *i-t* transients were removed to reduce the impact of the double layer charging current. All fits have R^2 values above 0.98 as can be seen in Figure S9d.

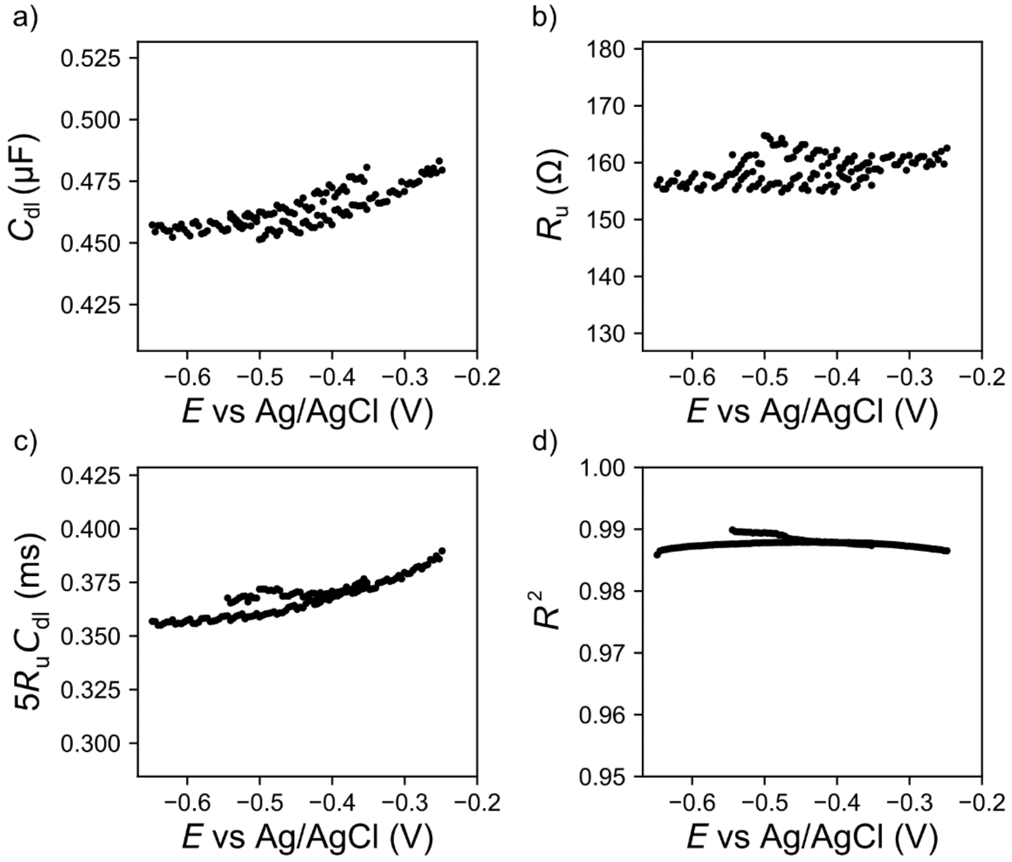

Figure S9. For the potential window between −0.6 and −0.2 V *vs* Ag/AgCl, using the BDD-Q pH electrode in 0.1 M KNO_3 at 250 Hz calculated (a) C_{dl}, (b) R_u, (c) $\times 5R_uC_{dl}$ and (d) the corresponding R^2 values to the fits using Equation S5. The parameters used for the SWV are the same as Figure S5.

SI 5. Additional Voltammetry of Cu^{2+}

Figure S10 shows the first scan CV recorded at a BDD electrode in an (a) deaerated and (b) aerated solution of 0.1 ppm Cu^{2+} in 0.1 M KNO_3 at pH 5.3 between −0.4 and 0.6 V *vs* Ag/AgCl at 0.1 V/s. The CV was scanned initially from 0.3 V to 0.6 V *vs* Ag/AgCl and cycled between +0.6 and -0.6 V at 0.1 V/s. The data is plotted for the first full scan.

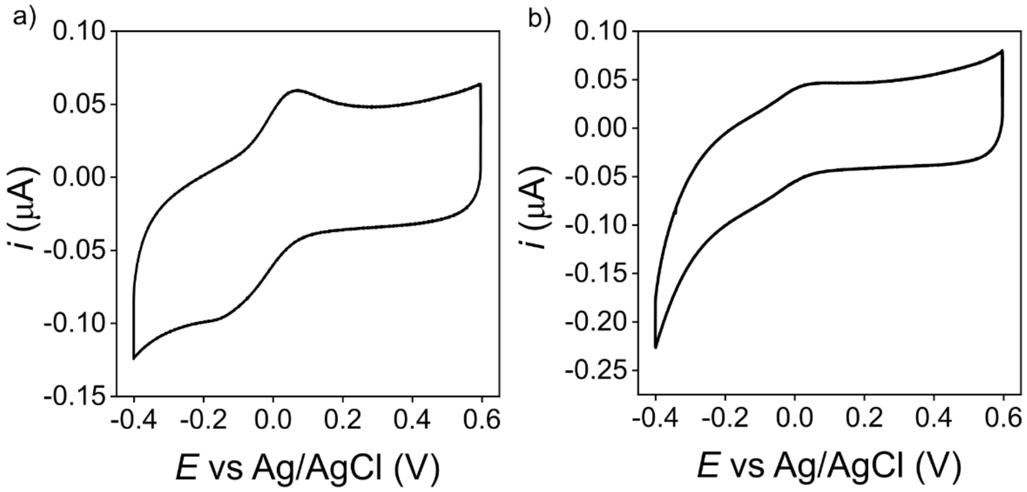

Figure S10. CVs of 0.1 ppm Cu^{2+} in 0.1 M KNO_3 at pH 5.3 when (a) purged with N_2 and (b) aerated, recorded at 0.1 V/s with a BDD electrode.

Figure S11a shows the i_{for} and reverse SWV waves when sampling the whole pulse between 1–100% corresponding to the data set used in Figure 4 of the main article. As the solution is not deaerated the large currents on the reverse wave between −0.1 to −0.4 V vs Ag/AgCl is attributed to oxygen reduction. Figure S11b shows i_{for} (red line) i_{rev} (blue line) and i_{diff}-t (black line) transients taken from the SWV *i-t* data at E = 0.061 V *vs* Ag/AgCl, recorded using a BDD electrode.

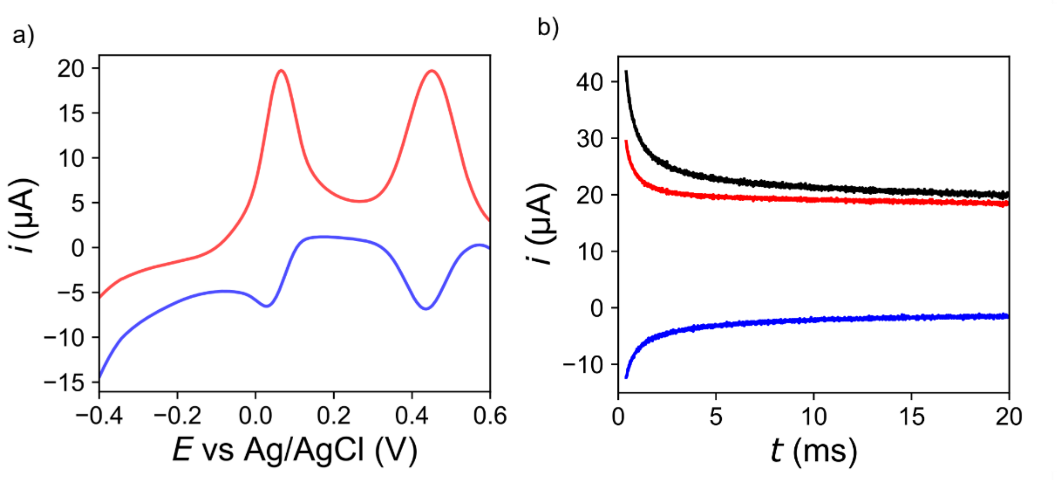

Figure S11. 400 µM Cu^{2+} and 100 µM $FcTMA^{+}$ in 0.1 M KNO3 at pH 5.8 (a) Plot of the i_{for} and i_{rev} (blue) SWVs when current averaging over 1–100%. (b) Plots of i_{for}-t (red line) i_{rev}-t (blue line) and i_{diff}-t (black line) transients at E = 0.061 V *vs* Ag/AgCl.

SI 6. CV response of BDD-Q Electrode

CV for the BDD-Q pH electrode in 0.1 M KNO_3 (pH 5.8) recorded at 1 V/s

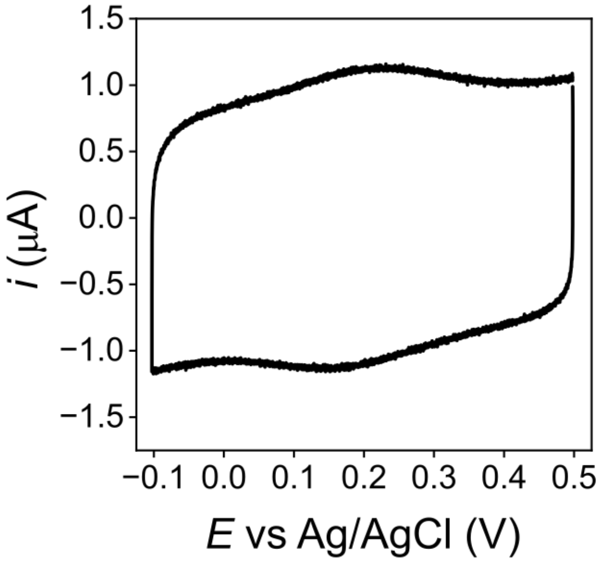

Figure S12. CV of a BDD-Q electrode in pH 5.8 in 0.1 M KNO_3 at 1 V/s.

SI 7. References

1 A. J. Bard, L. R. Faulkner and H. S. White, *Electrochemical Methods: Fundamentals and Applications*, Wiley, 3rd edn., 2022.

2 S. J. Cobb and J. V Macpherson, *Anal Chem*, 2019, **91**, 7935–7942.

3 Z. J. Ayres, S. J. Cobb, M. E. Newton and J. V. Macpherson, *Electrochem commun*, 2016, **72**, 59–63.
