# Supplementary material for: A Current Averaging Strategy for Maximizing Analyte and Minimizing Redox Interference Signals with Square Wave Voltammetry
Source: Anal Chem. 2024 May 26;96(23):9561–9. doi: 10.1021/acs.analchem.4c01053 (PMC11170553; doi:10.1021/acs.analchem.4c01053)

COMSOL Generated Report for Modelling SWV of an Outer-Sphere Redox Couple

Report date	Jan 29, 2024, 2:21:32 PM

Contents

1. Global Definitions 3
1.1. Parameters 3
1.2. Shared Properties 4
2. Component 1 5
2.1. Definitions 5
2.2. Geometry 1 11
2.3. Soluble Redox Species - Transport of Diluted Species 2 11
2.4. Events 18
2.5. Mesh 1 23
3. Study 1 27
3.1. Parametric Sweep 27
3.2. Stationary 27
3.3. Time Dependent 28
3.4. Solver Configurations 28
4. Results 33
4.1. Datasets 33
4.2. Derived Values 34
4.3. Tables .... **Error! Bookmark not defined.**
4.4. Plot Groups 35

1 Global Definitions

Date	Oct 18, 2023, 2:20:47 PM

GLOBAL SETTINGS

Version	COMSOL Multiphysics 6.1 (Build: 357)

USED PRODUCTS

COMSOL Multiphysics
Chemical Reaction Engineering Module

COMPUTER INFORMATION

CPU	Intel64 Family 6 Model 158 Stepping 9, 4 cores, 63.88 GB RAM
Operating system	Windows 10

1.1 PARAMETERS

1.1.1 Soluble Redox Species

SOLUBLE REDOX SPECIES

Name	Expression	Value	Description
DcOx	6.7E-06[cm^2/s]	6.7E−10 m^2/s	Diffusion Coefficient Oxidised species
DcRed	6.7E-06[cm^2/s]	6.7E−10 m^2/s	Diffusion Coefficient reduced species
cOx0_dif	1 [mM]	1 mol/m^3	Initial concentration of Ox species
k0_Dif	5 [cm/s]	0.05 m/s	Rate constant (fast)
alpha_Dif	0.5	0.5	Transfer coefficient
E0_Dif	0.43 [V]	0.43 V	Standard redox potential for [Ru(NH3)6]+3/+2 Vs SCE
n	1	1	Number of electrons transferred

1.1.2 Genral System & Geometry

GENRAL SYSTEM & GEOMETRY

Name	Expression	Value	Description
L	0.25[cm]	0.0025 m	Length of domain/boundary
nF_RT	(n*F_const)/(R_const*T)	39.586 1/V	
T	293.15[K]	293.15 K	Temperature
f	F_const/(R_const*T)	39.586 1/V	
A	((1.5 [mm])^2)*pi	7.0686E−6 m^2	Area for a 3 mm diameter electrode

1.1.3 Waveform

WAVEFORM

Name	Expression	Value	Description
v	1 [V/s]	1 V/s	Scan rate
tau	1/(2*f_SWV)	0.02 s	Length of one half cycle 2*tau = 1/fsw
f_SWV	25[Hz]	25 Hz	SWV frequency
E_Start	0.2[V]	0.2 V	Starting potential applied to the working electrode
E_Low	-0.5 [V]	−0.5 V	Switching overpotential
E_I	10[mV]	0.01 V	Potential increment of the linear ramp
E_High	0.1 [V]	0.1 V	Initial overpotential
E_Amp	50[mV]	0.05 V	SWV Amplitude

1.2 SHARED PROPERTIES

1.2.1 Default Model Inputs

Tag	cminpt

2 Component 1

SETTINGS

Description	Value
Unit system	Same as global system (SI)

2.1 DEFINITIONS

2.1.1 Variables

Butler-Volmer kinetics

SELECTION

Geometric entity level	Boundary
Selection	Geometry geom1: Dimension 0: Boundary 1

Name	Expression	Unit	Description
kf_Dif	k0_Dif*exp((-alpha_Dif*f*(Potential(t) - E0_Dif)))	m/s	
kb_Dif	k0_Dif*exp(((1 - alpha_Dif)*f*(Potential(t) - E0_Dif)))	m/s	

2.1.2 Functions

Potential waveform function

Function name	Potential
Function type	Piecewise

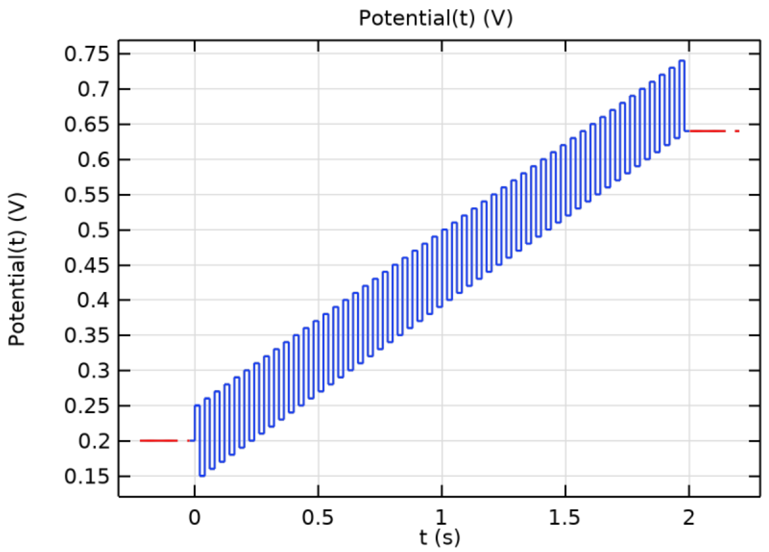

Potential waveform function

DEFINITION

Description	Value
Argument	t
Extrapolation	Constant
Smoothing	No smoothing

DEFINITION

Start	End	Function
-tau	0	E_Start
0	tau	E_Start+E_Amp
tau	tau*2	E_Start-E_Amp
tau*2	tau*3	E_Start+E_Amp+E_I*1
tau*3	tau*4	E_Start-E_Amp+E_I*1
tau*4	tau*5	E_Start+E_Amp+E_I*2
tau*5	tau*6	E_Start-E_Amp+E_I*2
tau*6	tau*7	E_Start+E_Amp+E_I*3
tau*7	tau*8	E_Start-E_Amp+E_I*3
tau*8	tau*9	E_Start+E_Amp+E_I*4
tau*9	tau*10	E_Start-E_Amp+E_I*4
tau*10	tau*11	E_Start+E_Amp+E_I*5
tau*11	tau*12	E_Start-E_Amp+E_I*5
tau*12	tau*13	E_Start+E_Amp+E_I*6
tau*13	tau*14	E_Start-E_Amp+E_I*6
tau*14	tau*15	E_Start+E_Amp+E_I*7
tau*15	tau*16	E_Start-E_Amp+E_I*7
tau*16	tau*17	E_Start+E_Amp+E_I*8
tau*17	tau*18	E_Start-E_Amp+E_I*8
tau*18	tau*19	E_Start+E_Amp+E_I*9
tau*19	tau*20	E_Start-E_Amp+E_I*9
tau*20	tau*21	E_Start+E_Amp+E_I*10
tau*21	tau*22	E_Start-E_Amp+E_I*10
tau*22	tau*23	E_Start+E_Amp+E_I*11
tau*23	tau*24	E_Start-E_Amp+E_I*11
tau*24	tau*25	E_Start+E_Amp+E_I*12
tau*25	tau*26	E_Start-E_Amp+E_I*12
tau*26	tau*27	E_Start+E_Amp+E_I*13
tau*27	tau*28	E_Start-E_Amp+E_I*13

Start	End	Function
tau*28	tau*29	E_Start+E_Amp+E_I*14
tau*29	tau*30	E_Start-E_Amp+E_I*14
tau*30	tau*31	E_Start+E_Amp+E_I*15
tau*31	tau*32	E_Start-E_Amp+E_I*15
tau*32	tau*33	E_Start+E_Amp+E_I*16
tau*33	tau*34	E_Start-E_Amp+E_I*16
tau*34	tau*35	E_Start+E_Amp+E_I*17
tau*35	tau*36	E_Start-E_Amp+E_I*17
tau*36	tau*37	E_Start+E_Amp+E_I*18
tau*37	tau*38	E_Start-E_Amp+E_I*18
tau*38	tau*39	E_Start+E_Amp+E_I*19
tau*39	tau*40	E_Start-E_Amp+E_I*19
tau*40	tau*41	E_Start+E_Amp+E_I*20
tau*41	tau*42	E_Start-E_Amp+E_I*20
tau*42	tau*43	E_Start+E_Amp+E_I*21
tau*43	tau*44	E_Start-E_Amp+E_I*21
tau*44	tau*45	E_Start+E_Amp+E_I*22
tau*45	tau*46	E_Start-E_Amp+E_I*22
tau*46	tau*47	E_Start+E_Amp+E_I*23
tau*47	tau*48	E_Start-E_Amp+E_I*23
tau*48	tau*49	E_Start+E_Amp+E_I*24
tau*49	tau*50	E_Start-E_Amp+E_I*24
tau*50	tau*51	E_Start+E_Amp+E_I*25
tau*51	tau*52	E_Start-E_Amp+E_I*25
tau*52	tau*53	E_Start+E_Amp+E_I*26
tau*53	tau*54	E_Start-E_Amp+E_I*26
tau*54	tau*55	E_Start+E_Amp+E_I*27
tau*55	tau*56	E_Start-E_Amp+E_I*27
tau*56	tau*57	E_Start+E_Amp+E_I*28
tau*57	tau*58	E_Start-E_Amp+E_I*28
tau*58	tau*59	E_Start+E_Amp+E_I*29
tau*59	tau*60	E_Start-E_Amp+E_I*29
tau*60	tau*61	E_Start+E_Amp+E_I*30
tau*61	tau*62	E_Start-E_Amp+E_I*30
tau*62	tau*63	E_Start+E_Amp+E_I*31

Start	End	Function
tau*63	tau*64	E_Start-E_Amp+E_I*31
tau*64	tau*65	E_Start+E_Amp+E_I*32
tau*65	tau*66	E_Start-E_Amp+E_I*32
tau*66	tau*67	E_Start+E_Amp+E_I*33
tau*67	tau*68	E_Start-E_Amp+E_I*33
tau*68	tau*69	E_Start+E_Amp+E_I*34
tau*69	tau*70	E_Start-E_Amp+E_I*34
tau*70	tau*71	E_Start+E_Amp+E_I*35
tau*71	tau*72	E_Start-E_Amp+E_I*35
tau*72	tau*73	E_Start+E_Amp+E_I*36
tau*73	tau*74	E_Start-E_Amp+E_I*36
tau*74	tau*75	E_Start+E_Amp+E_I*37
tau*75	tau*76	E_Start-E_Amp+E_I*37
tau*76	tau*77	E_Start+E_Amp+E_I*38
tau*77	tau*78	E_Start-E_Amp+E_I*38
tau*78	tau*79	E_Start+E_Amp+E_I*39
tau*79	tau*80	E_Start-E_Amp+E_I*39
tau*80	tau*81	E_Start+E_Amp+E_I*40
tau*81	tau*82	E_Start-E_Amp+E_I*40
tau*82	tau*83	E_Start+E_Amp+E_I*41
tau*83	tau*84	E_Start-E_Amp+E_I*41
tau*84	tau*85	E_Start+E_Amp+E_I*42
tau*85	tau*86	E_Start-E_Amp+E_I*42
tau*86	tau*87	E_Start+E_Amp+E_I*43
tau*87	tau*88	E_Start-E_Amp+E_I*43
tau*88	tau*89	E_Start+E_Amp+E_I*44
tau*89	tau*90	E_Start-E_Amp+E_I*44
tau*90	tau*91	E_Start+E_Amp+E_I*45
tau*91	tau*92	E_Start-E_Amp+E_I*45
tau*92	tau*93	E_Start+E_Amp+E_I*46
tau*93	tau*94	E_Start-E_Amp+E_I*46
tau*94	tau*95	E_Start+E_Amp+E_I*47
tau*95	tau*96	E_Start-E_Amp+E_I*47
tau*96	tau*97	E_Start+E_Amp+E_I*48
tau*97	tau*98	E_Start-E_Amp+E_I*48

Start	End	Function
tau*98	tau*99	E_Start+E_Amp+E_I*49
tau*99	tau*100	E_Start-E_Amp+E_I*49

UNITS

Description	Value
Arguments	s
Function	V

2.1.3 Probes

Total Current Probe

Probe type	Point probe

SELECTION

Geometric entity level	Boundary
Selection	Geometry geom1: Dimension 0: Boundary 1

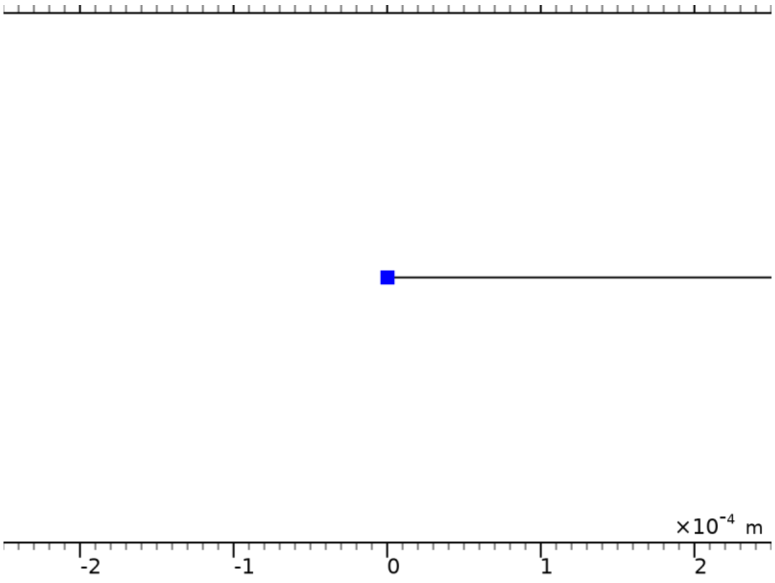

Selection

PROBE TYPE

Description	Value
Type	Integral

EXPRESSION

Description	Value
Expression	(-(tds2.ntflux_cOx_dif)*F_const*n)

Description	Value
Table and plot unit	mA/cm^2
Description	(-(tds2.ntflux_cOx_dif)*F_const*n)

INTEGRATION SETTINGS

Description	Value
Frame	Spatial

TABLE AND WINDOW SETTINGS

Description	Value
Output table	Probe Table 8
Plot window	Probe Plot 2

2.1.4 Nonlocal Couplings

Integration 1

Coupling type	Integration
Operator name	intop1

SELECTION

Geometric entity level	Boundary
Selection	Geometry geom1: Dimension 0: Boundary 1

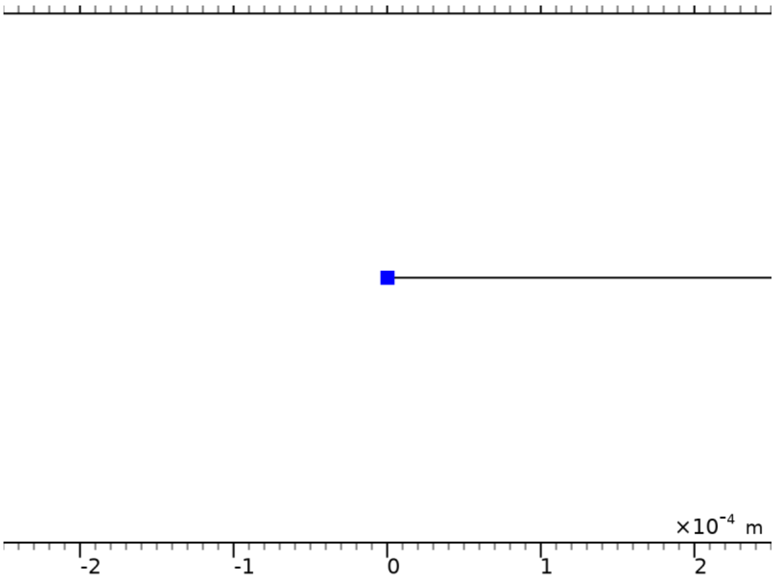

Selection

2.2 GEOMETRY 1

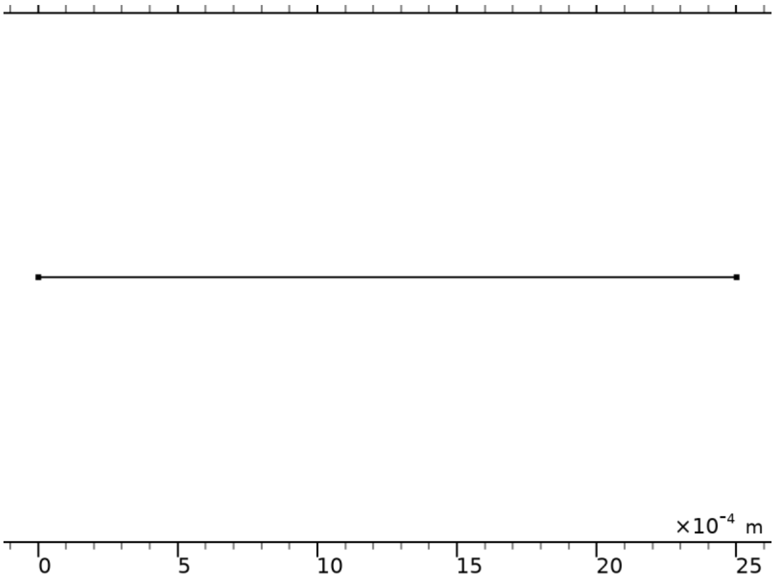

Geometry 1

UNITS

Length unit	m
Angular unit	deg

GEOMETRY STATISTICS

Description	Value
Space dimension	1
Number of domains	1
Number of boundaries	2

2.2.1 Interval 1 (i1)

INTERVAL

Coordinates (m)
0
L

2.3 SOLUBLE REDOX SPECIES - TRANSPORT OF DILUTED SPECIES 2

USED PRODUCTS

COMSOL Multiphysics
Chemical Reaction Engineering Module

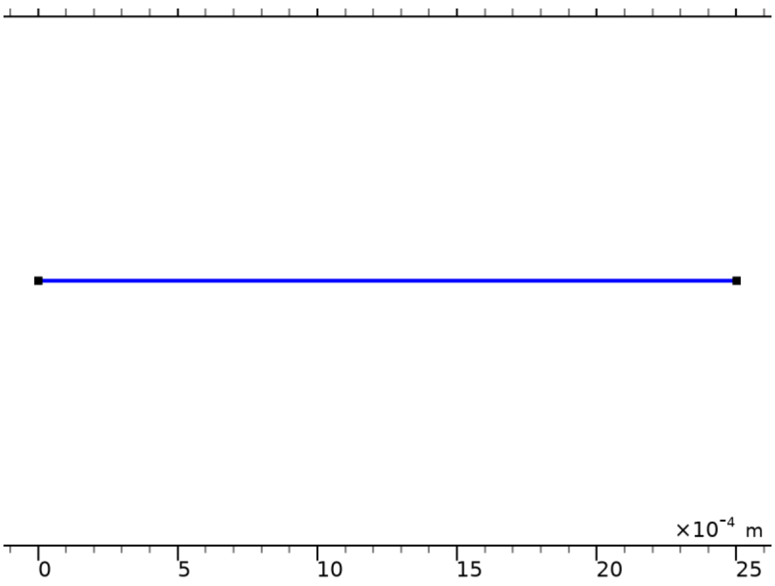

Soluble Redox Species - Transport of Diluted Species 2

SELECTION

Geometric entity level	Domain
Selection	Geometry geom1: Dimension 1: Domain 1

EQUATIONS

$$\nabla \cdot \mathbf{J}_i = R_i$$
$$\mathbf{J}_i = -D_i \nabla c_i$$

2.3.1 Interface Settings

Discretization

SETTINGS

**Description**	**Value**
Concentration	Linear

SETTINGS

**Description**	**Value**
Equation form	Study controlled

Cross-Sectional Area

SETTINGS

**Description**	**Value**	**Unit**
Cross-sectional area	A	m^2

Species Activity

SETTINGS

Description	Value
Species activity	Ideal

Transport Mechanisms

SETTINGS

Description	Value
Convection	Off
Migration in electric field	Off
Mass transfer in porous media	Off

2.3.2 Transport Properties 1

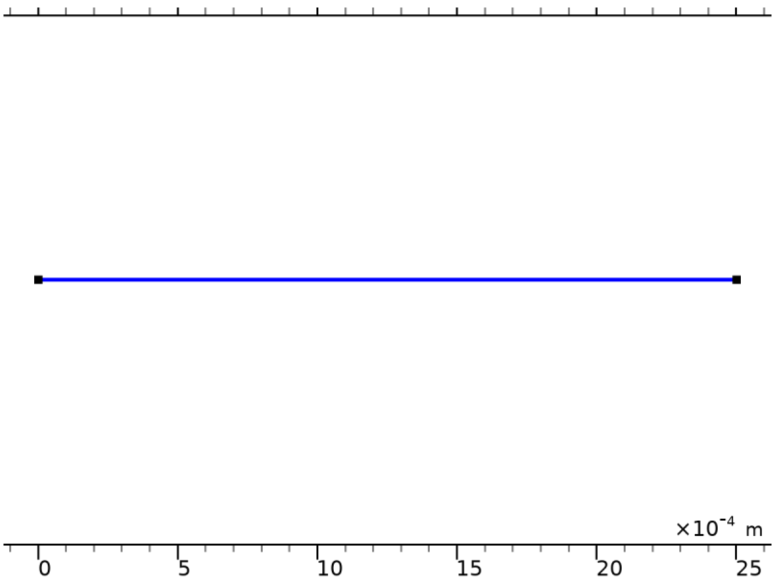

Transport Properties 1

SELECTION

Geometric entity level	Domain
Selection	Geometry geom1: Dimension 1: All domains

EQUATIONS

$$\nabla \cdot \mathbf{J}_i = R_i$$
$$\mathbf{J}_i = -D_i \nabla c_i$$

Diffusion

SETTINGS

Description	Value	Unit
Source	Material	
Material	None	
Diffusion coefficient	User defined	
Diffusion coefficient	DcOx	m^2/s
Diffusion coefficient	User defined	
Diffusion coefficient	DcRed	m^2/s

Coordinate System Selection

SETTINGS

Description	Value
Coordinate system	Global coordinate system

Model Input

SETTINGS

Description	Value	Unit
Temperature	User defined	
Temperature	T	K

Shape functions

Name	Shape function	Unit	Description	Shape frame	Selection
cOx_dif	Lagrange (Linear)	mol/m^3	Concentration	Spatial	Domain 1
cRed_dif	Lagrange (Linear)	mol/m^3	Concentration	Spatial	Domain 1

Weak Expressions

Weak expression	Integration order	Integration frame	Selection
tds2.dflux_cOx_difx*test(cOx_difx)*tds2.d	2	Spatial	Domain 1
tds2.dflux_cRed_difx*test(cRed_difx)*tds2.d	2	Spatial	Domain 1
tds2.streamline*(isScalingSystemDomain==0)*tds2.d	2	Spatial	Domain 1
tds2.crosswind*(isScalingSystemDomain==0)*tds2.d	4	Spatial	Domain 1

2.3.3 No Flux 1

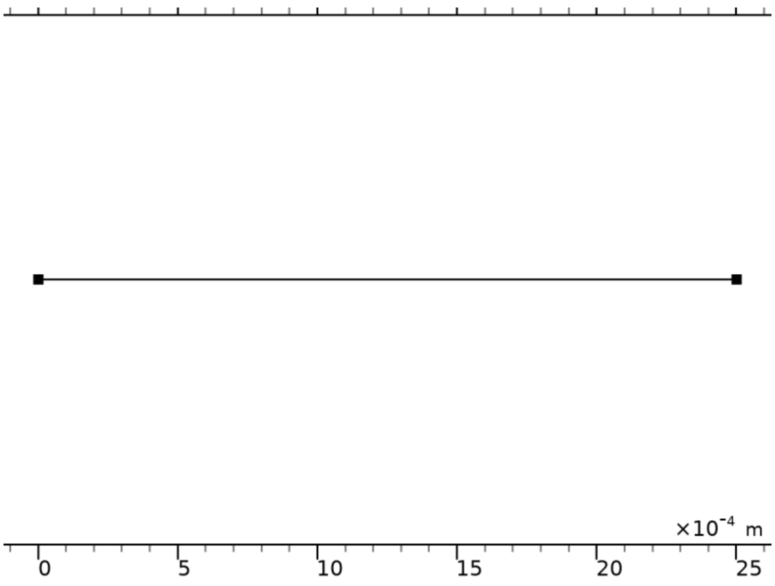

No Flux 1

SELECTION

Geometric entity level	Boundary
Selection	Geometry geom1: Dimension 0: All boundaries

EQUATIONS

$$-\mathbf{n}\cdot\mathbf{J}_i=0$$

2.3.4 Initial Values 1

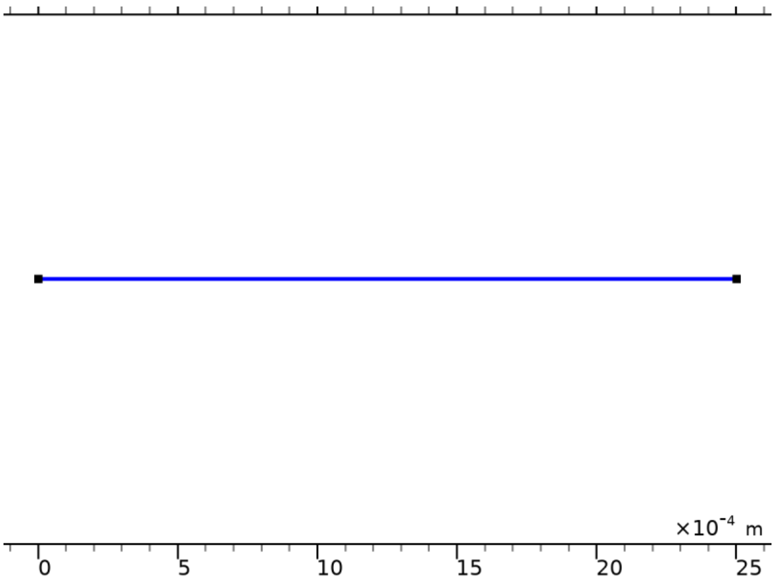

Initial Values 1

SELECTION

Geometric entity level	Domain
Selection	Geometry geom1: Dimension 1: All domains

Initial Values

SETTINGS

Description	Value	Unit
Concentration	{0, cOx0_dif}	mol/m³

Variables

Name	Expression	Unit	Description	Selection	Details
tds2.c0_cOx_dif	0	mol/m³	Concentration	Domain 1	+ operation
tds2.c0_cRed_dif	cOx0_dif	mol/m³	Concentration	Domain 1	+ operation

2.3.5 Concentration 1

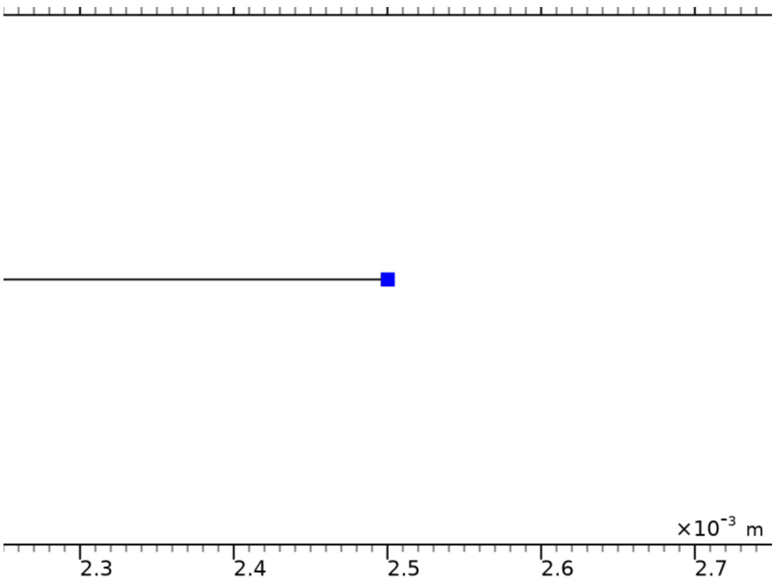

Concentration 1

SELECTION

Geometric entity level	Boundary
Selection	Geometry geom1: Dimension 0: Boundary 2

EQUATIONS

$$c_i = c_{0,i}$$

Concentration

SETTINGS

Description	Value	Unit
Species cOx_dif	On	
Species cRed_dif	On	
Concentration	{0, root.cOx0_dif}	mol/m³

Variables

Name	Expression	Unit	Description	Selection	Details
tds2.c0_cOx_dif	0	mol/m³	Concentration	Boundary 2	+ operation
tds2.c0_cRed_dif	cOx0_dif	mol/m³	Concentration	Boundary 2	+ operation
tds2.conc1.nmflow_cOx_dif	tds2.conc1.int(tds2.ntflux_cOx_dif)*tds2.d	mol/s	Normal molar flow rate	Global	
tds2.conc1.nmflow_cRed_dif	tds2.conc1.int(tds2.ntflux_cRed_dif)*tds2.d	mol/s	Normal molar flow rate	Global	

Constraints

Constraint	Constraint force	Shape function	Selection	Details
-tds2.cVar_cOx_dif+tds2.c0_cOx_dif	test(-tds2.cVar_cOx_dif+tds2.c0_cOx_dif)	Lagrange (Linear)	Boundary 2	Elemental
-tds2.cVar_cRed_dif+tds2.c0_cRed_dif	test(-tds2.cVar_cRed_dif+tds2.c0_cRed_dif)	Lagrange (Linear)	Boundary 2	Elemental

2.3.6 Butler Volmer HET

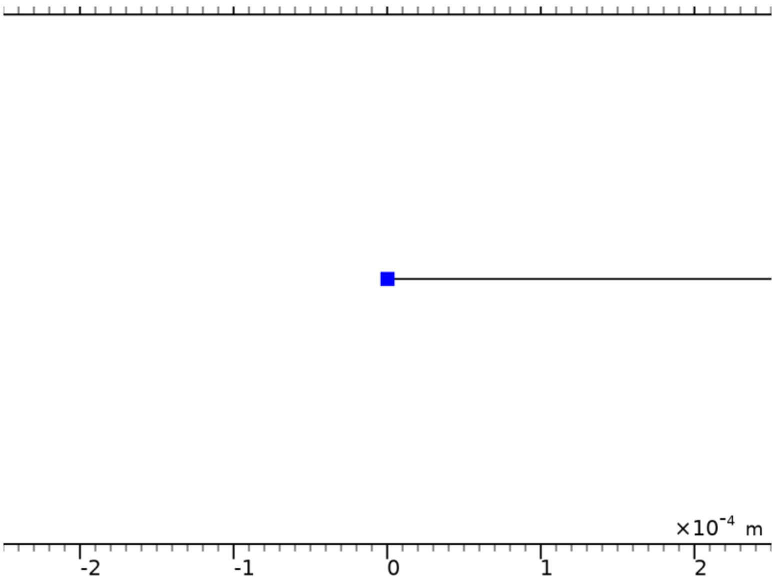

Butler Volmer HET

SELECTION

Geometric entity level	Boundary
Selection	Geometry geom1: Dimension 0: Boundary 1

EQUATIONS

$$-\mathbf{n}\cdot\mathbf{J}_i = J_{0,i}$$

Inward Flux

SETTINGS

Description	Value	Unit
Flux type	General inward flux	
Species cOx_dif	On	
Species cRed_dif	On	
	{-kf_Dif*cOx_dif + kb_Dif*cRed_dif, kf_Dif*cOx_dif - kb_Dif*cRed_dif}	mol/(m²·s)

Variables

Name	Expression	Unit	Description	Selection
tds2.fl1.nmflow_cOx_dif	tds2.fl1.int(tds2.ntflux_cOx_dif)*tds2.d	mol/s	Normal molar flow rate	Global
tds2.fl1.nmflow_cRed_dif	tds2.fl1.int(tds2.ntflux_cRed_dif)*tds2.d	mol/s	Normal molar flow rate	Global

Weak Expressions

Weak expression	Integration order	Integration frame	Selection
(-kf_Dif*cOx_dif+kb_Dif*cRed_dif)*test(cOx_dif)*tds2.d	2	Spatial	Boundary 1
-(-kf_Dif*cOx_dif+kb_Dif*cRed_dif)*test(cRed_dif)*tds2.d	2	Spatial	Boundary 1

2.4 EVENTS

USED PRODUCTS

COMSOL Multiphysics

SELECTION

Geometric entity level	Domain
Selection	Geometry geom1: Dimension 1: All domains

2.4.1 Interface Settings

Discretization

SETTINGS

Description	Value
Element order	Quadratic

SETTINGS

Description	Value
Equation form	Study controlled

2.4.2 Explicit Event 1

SELECTION

Geometric entity level	Entire model

Event Timings

SETTINGS

Description	Value	Unit
Start of event	-1.0E-5 + tau	s
Period of event	Inf	s
Use consistent initialization	On	

Reinitialization

SETTINGS

Description	Value
Variable	

2.4.3 Explicit Event 2

SELECTION

Geometric entity level	Entire model

Event Timings

SETTINGS

Description	Value	Unit
Start of event	2*tau	s
Period of event	Inf	s
Use consistent initialization	On	

Reinitialization

SETTINGS

Description	Value
Variable	

2.4.4 Explicit Event 3

SELECTION

Geometric entity level	Entire model

Event Timings

SETTINGS

Description	Value	Unit
Start of event	3*tau	s
Period of event	Inf	s
Use consistent initialization	On	

Reinitialization

SETTINGS

Description	Value
Variable	

2.4.5 Explicit Event 4

SELECTION

Geometric entity level	Entire model

Event Timings

SETTINGS

Description	Value	Unit
Start of event	4*tau	s
Period of event	Inf	s
Use consistent initialization	On	

Reinitialization

SETTINGS

Description	Value
Variable	

2.4.6 Explicit Event 5

SELECTION

Geometric entity level	Entire model

Event Timings

SETTINGS

Description	Value	Unit
Start of event	5*tau	s
Period of event	Inf	s
Use consistent initialization	On	

Reinitialization

SETTINGS

Description	Value
Variable	

2.4.7 Explicit Event 6

SELECTION

Geometric entity level	Entire model

Event Timings

SETTINGS

Description	Value	Unit
Start of event	6*tau	s
Period of event	Inf	s
Use consistent initialization	On	

Reinitialization

SETTINGS

Description	Value
Variable	

2.4.8 Explicit Event 7

SELECTION

Geometric entity level	Entire model

Event Timings

SETTINGS

Description	Value	Unit
Start of event	7*tau	s
Period of event	Inf	s
Use consistent initialization	On	

Reinitialization

SETTINGS

Description	Value
Variable	

2.4.9 Explicit Event 8

SELECTION

Geometric entity level	Entire model

Event Timings

SETTINGS

Description	Value	Unit
Start of event	8*tau	s
Period of event	Inf	s
Use consistent initialization	On	

Reinitialization

SETTINGS

Description	Value
Variable	

2.4.10 Explicit Event 9

SELECTION

Geometric entity level	Entire model

Event Timings

SETTINGS

Description	Value	Unit
Start of event	9*tau	s
Period of event	Inf	s
Use consistent initialization	On	

Reinitialization

SETTINGS

Description	Value
Variable	

2.4.11 Explicit Event 10

SELECTION

Geometric entity level	Entire model

Event Timings

SETTINGS

Description	Value	Unit
Start of event	10*tau	s
Period of event	Inf	s
Use consistent initialization	On	

Reinitialization

SETTINGS

Description	Value
Variable	

**** Note all 100 potential steps each have an explicit event defined but 11 – 100 have been removed to reduce the file size of the mode report ****

2.5 MESH 1

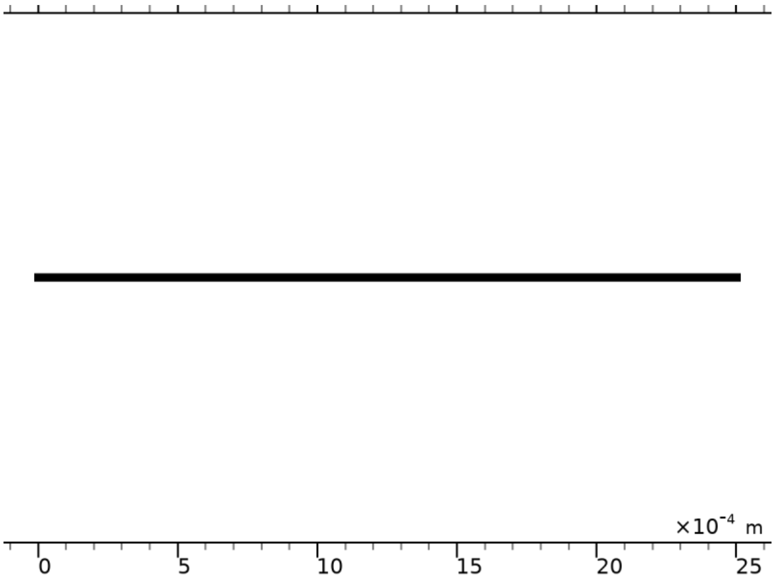

Mesh 1

2.5.1 Size (size)

SETTINGS

Description	Value
Maximum element size	L/100
Minimum element size	3E-8
Curvature factor	0.3
Maximum element growth rate	1.3
Custom element size	Custom

2.5.2 Size 1 (size1)

SELECTION

Geometric entity level	Boundary
Selection	Geometry geom1: Dimension 0: Boundary 1

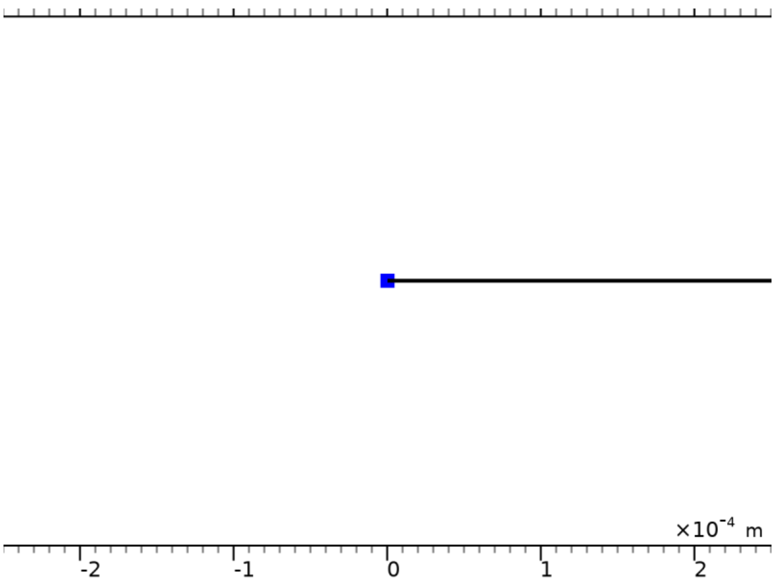

Size 1

SETTINGS

Description	Value
Maximum element size	0.3[nm]
Minimum element size	3E-8
Minimum element size	Off
Curvature factor	0.3
Curvature factor	Off
Resolution of narrow regions	Off
Custom element size	Custom

2.5.3 Edge 1 (edg1)

SELECTION

Geometric entity level	Domain
Selection	Geometry geom1: Dimension 1: Domain 1

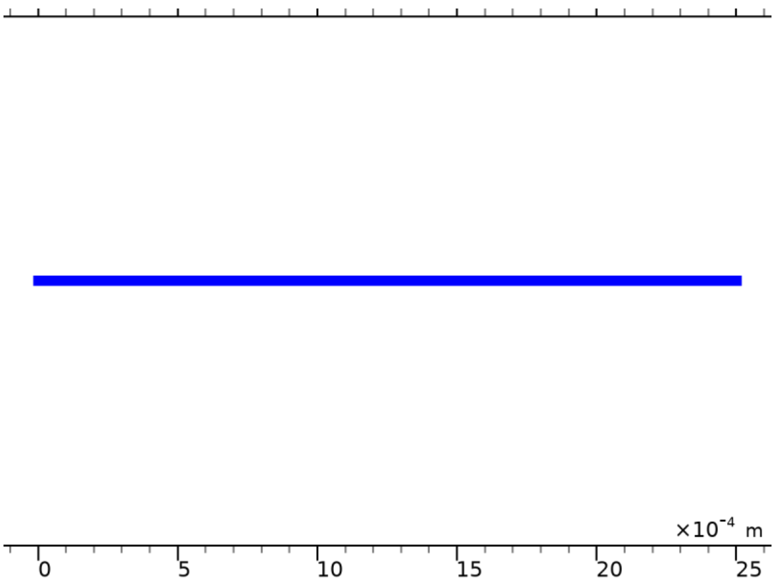

Edge 1

SETTINGS

Description	Value
Last build time	0
Built with	COMSOL 6.1.0.357 (win64)|2024 - 01 - 25T19:12:52.072646700

3 Study 1

COMPUTATION INFORMATION

Computation time	57 min 24 s

3.1 PARAMETRIC SWEEP

Parameter name	Parameter value list	Parameter unit
k0_Dif	5	cm/s

STUDY SETTINGS

Description	Value
Sweep type	Specified combinations
Parameter name	k0_Dif
Unit	cm/s

PARAMETERS

Parameter name	Parameter value list	Parameter unit
k0_Dif (Rate constant (fast))	5	cm/s

3.2 STATIONARY

STUDY SETTINGS

Description	Value
Include geometric nonlinearity	Off

PHYSICS AND VARIABLES SELECTION

Physics interface	Solve for	Equation form
Soluble Redox Species - Transport of Diluted Species 2 (tds2)	On	Automatic (Stationary)
Events (ev)	Off	Automatic (Time dependent)

VALUES OF DEPENDENT VARIABLES

Description	Value
Settings	User controlled

MESH SELECTION

Component	Mesh
Component 1	Mesh 1

3.3 TIME DEPENDENT

Times	Unit
-tau, range(0,1.0e-5,tau*90)	s

STUDY SETTINGS

Description	Value
Include geometric nonlinearity	Off

PHYSICS AND VARIABLES SELECTION

Physics interface	Solve for	Equation form
Soluble Redox Species - Transport of Diluted Species 2 (tds2)	On	Automatic (Time dependent)
Events (ev)	On	Automatic (Time dependent)

VALUES OF DEPENDENT VARIABLES

Description	Value
Settings	User controlled
Method	Solution
Study	Study 1

MESH SELECTION

Component	Mesh
Component 1	Mesh 1

3.4 SOLVER CONFIGURATIONS

3.4.1 Solution 2

Compile Equations: Stationary (st1)

STUDY AND STEP

Description	Value
Use study	Study 1
Use study step	Stationary

Dependent Variables 1 (v1)

GENERAL

Description	Value
Defined by study step	Stationary

INITIAL VALUE CALCULATION CONSTANTS

Constant name	Initial value source
t	0
timestep	0.00182[s]

Concentration (comp1.cOx_dif) (comp1_cOx_dif)

GENERAL

Description	Value
Field components	comp1.cOx_dif
Internal variables	{comp1.uflux.cOx_dif, comp1.dflux.cOx_dif}

Concentration (comp1.cRed_dif) (comp1_cRed_dif)

GENERAL

Description	Value
Field components	comp1.cRed_dif
Internal variables	{comp1.uflux.cRed_dif, comp1.dflux.cRed_dif}

Stationary Solver 1 (s1)

GENERAL

Description	Value
Defined by study step	Stationary

RESULTS WHILE SOLVING

Description	Value
Probes	None

CONSTANTS

Constant name	Constant value
t	0

Advanced (aDef)

ASSEMBLY SETTINGS

Description	Value
Reuse sparsity pattern	On

Fully Coupled 1 (fc1)

GENERAL

Description	Value
Linear solver	Direct 1

METHOD AND TERMINATION

Description	Value
Initial damping factor	0.01
Minimum damping factor	1E-6
Maximum number of iterations	50

Direct 1 (d1)

GENERAL

Description	Value
Solver	PARDISO
Pivoting perturbation	1E-13

Solution Store 1 (su1)

GENERAL

Description	Value
Solution	Solution Store 1

Compile Equations: Time Dependent (st2)

STUDY AND STEP

Description	Value
Use study	Study 1
Use study step	Time Dependent

Dependent Variables 2 (v2)

GENERAL

Description	Value
Defined by study step	Time Dependent

INITIAL VALUES OF VARIABLES SOLVED FOR

Description	Value
Method	Solution
Solution	Solution 2

RESIDUAL SCALING

Description	Value
Method	Manual

VALUES OF VARIABLES NOT SOLVED FOR

Description	Value
Method	Solution

Description	Value
Solution	Solution 2

INITIAL VALUE CALCULATION CONSTANTS

Constant name	Initial value source
t	-tau, range(0,1.0e-5,tau*90)
timestep	0.00182[s]

Concentration (comp1.cOx_dif) (comp1_cOx_dif)

GENERAL

Description	Value
Field components	comp1.cOx_dif
Internal variables	{comp1.uflux.cOx_dif, comp1.dflux.cOx_dif, comp1.tds2.dt2Inv_cOx_dif}

Concentration (comp1.cRed_dif) (comp1_cRed_dif)

GENERAL

Description	Value
Field components	comp1.cRed_dif
Internal variables	{comp1.uflux.cRed_dif, comp1.dflux.cRed_dif, comp1.tds2.dt2Inv_cRed_dif}

Time-Dependent Solver 1 (t1)

ABSOLUTE TOLERANCE

Field	Method	Tolerance method	Tolerance factor	Derivative tolerance method	Tolerance for time derivatives	Tolerance	Tolerance for time derivatives
Concentration (comp1.cOx_dif)	Use global	Factor	0.1	Automatic	1	0.001	0.001
Concentration (comp1.cRed_dif)	Use global	Factor	0.1	Automatic	1	0.001	0.001

TIME STEPPING

Description	Value
Steps taken by solver	Strict
Maximum BDF order	2
Nonlinear controller	On

Advanced (aDef)

ASSEMBLY SETTINGS

Description	Value
Reuse sparsity pattern	On

Fully Coupled 1 (fc1)

GENERAL

Description	Value
Linear solver	Direct 1

METHOD AND TERMINATION

Description	Value
Nonlinear method	Automatic (Newton)
Initial damping factor	0.9
Restriction for step-size update	50
Maximum number of iterations	100

3.4.2 Parametric Solutions 1

k0_Dif=5 (su1)

GENERAL

Description	Value
Solution	k0_Dif=5

4 Results

4.1 DATASETS

4.1.1 Study 1/Solution 2

SOLUTION

Description	Value
Solution	Solution 2
Component	Component 1 (comp1)
Frame	Spatial (x, y, z)

4.1.2 Study 1/Parametric Solutions 1

SOLUTION

Description	Value
Solution	Parametric Solutions 1
Component	Component 1 (comp1)
Frame	Spatial (x, y, z)

4.1.3 Grid 1D 1

DATA

Description	Value
Function	Potential waveform function
Refresh	

PARAMETER BOUNDS

Description	Value
Name	t
Minimum	-2
Maximum	22

4.1.4 Probe Solution 4

SOLUTION

Description	Value
Solution	Solution 2
Component	Component 1 (comp1)
Frame	Spatial (x, y, z)

4.1.5 Study 1/Solution Store 1

SOLUTION

Description	Value
Solution	Solution Store 1
Component	Component 1 (comp1)
Frame	Spatial (x, y, z)

4.1.6 Total Current Probe

SELECTION

Geometric entity level	Boundary
Selection	Geometry geom1: Dimension 0: Boundary 1

DATA

Description	Value
Dataset	Probe Solution 4

SETTINGS

Description	Value
Method	Integration
Integration order	4
Integration order	On

4.2 DERIVED VALUES

4.2.1 Total Current Probe

OUTPUT

Evaluated in	Probe Table 8

DATA

Description	Value
Dataset	Total Current Probe

EXPRESSIONS

Expression	Unit	Description
(-(tds2.ntflux_cOx_dif)*F_const*n)	mA/cm^2	

4.3 PLOT GROUPS

4.3.1 Current Density

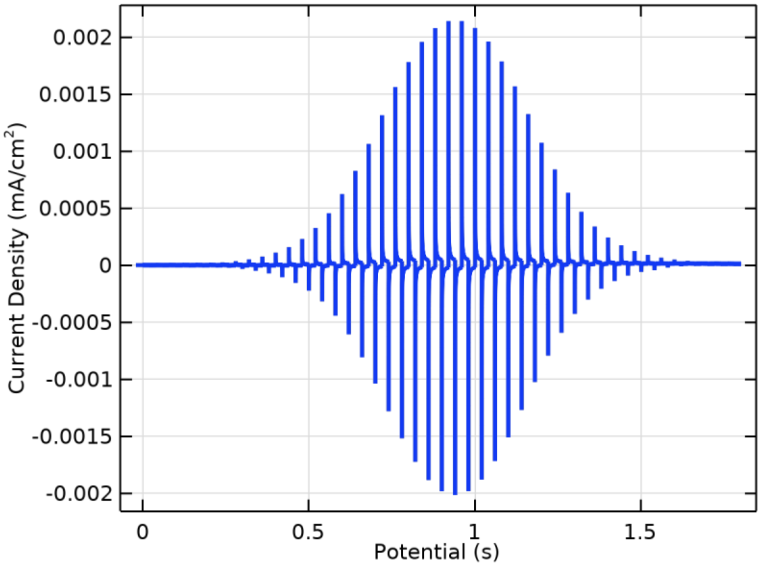

Supplement: Supplementary file 2 — ac4c01053_si_002.pdf [file ac4c01053_si_002.pdf]
